# ATM localization and gene expression in the adult mouse eye

**Published:** 2009-02-20

**Authors:** Julia Leemput, Christel Masson, Karine Bigot, Abdelmounaim Errachid, Anouk Dansault, Alexandra Provost, Stéphanie Gadin, Said Aoufouchi, Maurice Menasche, Marc Abitbol

**Affiliations:** 1 Université Paris-Descartes, CERTO, Centre de Recherche Thérapeutique en Ophtalmologie de la Faculté de Médecine Paris-Descartes-site Necker, Paris, France; 2 Université Paris-Descartes, Plateforme d’imagerie Cellulaire de la Faculté de Médecine Paris-Descartes-site Necker, Paris, France; 3 Université Paris-Descartes, Développement du Système Immunitaire, INSERM U783, Faculté de Médecine Paris-Descartes-site Necker, Paris, France

## Abstract

**Purpose:**

High levels of metabolism and oxygen consumption in most adult murine ocular compartments, combined with exposure to light and ultraviolet (UV) radiation, are major sources of oxidative stress, causing DNA damage in ocular cells. Of all mammalian body cells, photoreceptor cells consume the largest amount of oxygen and generate the highest levels of oxidative damage. The accumulation of such damage throughout life is a major factor of aging tissues. Several multiprotein complexes have recently been identified as the major sensors and mediators involved in the maintenance of DNA integrity. The activity of these complexes initially seemed to be restricted to dividing cells, given their ultimate role in major cell cycle checkpoints. However, it was later established that they are also active in post-mitotic cells. Recent findings demonstrate that the DNA damage response (DDR) is essential for the development, maintenance, and normal functioning of the adult central nervous system. One major molecular factor in the DDR is the protein, ataxia telangiectasia mutated (ATM). It is required for the rapid induction of cellular responses to DNA double-strand breaks. These cytotoxic DNA lesions may be caused by oxidative damage. To understand how ATM prevents oxidative stress and participates in the maintenance of genomic integrity and cell viability of the adult retina, we determined the ATM expression patterns and studied its localization in the adult mouse eye.

**Methods:**

*Atm* gene expression was analyzed by RT–PCR experiments and its localization by in situ hybridization on adult mouse ocular and cerebellar tissue sections. ATM protein expression was determined by western blot analysis of proteins homogenates extracted from several mouse tissues and its localization by immunohistochemistry experiments performed on adult mouse ocular and cerebellar tissue sections. In addition, subcellular localization was realized by confocal microscopy imaging of ocular tissue sections, with a special focus on retinal cells.

**Results:**

Using RT–PCR, we detected a band of the expected size, with its sequence matching the amplified *Atm* cDNA sequence. *Atm* mRNA was detected in most cell bodies of the adult mouse eye by in situ hybridization of ocular tissue sections with specific digoxigenin-labeled PCR-amplified cDNA probes. Western blotting with different specific antibodies revealed bands corresponding to the expected sizes of ATM and its active forms (ATMp). These bands were not observed in the analysis of protein homogenates from *Atm*-deficient mouse tissues. ATM immunoreactivity was detected in the nucleus of all adult mice retinal cells and in most non-neuronal ocular cell types. The active phosphorylated form of ATM was also present in the retina as well as in non-neuronal cells of the adult mouse eye. However, its subcellular localization differed as a function of the cell type examined. A major finding of this study was that ATMp immunostaining in photoreceptor cells was exclusively in the cytoplasm, whereas ATM immunostaining was only in the nucleus of these cells. Furthermore, the specific and distinct ATM and ATMp immunolabeling patterns in photoreceptor cells were identical to those observed in the adult mouse cerebellar granule cells.

**Conclusions:**

We report the expression profile of *Atm* gene and protein in the adult mouse eye. In particular, we observed a difference between the localization patterns of the active and inactive forms of ATM in photoreceptor cells. These localization patterns suggest that ATM and its phosphorylated activated form may be involved in both the protection of cells from oxidative damage and the maintenance of ocular cell structure and function. The protection mechanisms mediated by the two forms of ATM appear to be particularly important in maintaining photoreceptor integrity.

## Introduction

The retina is a part of the central nervous system (CNS). It forms from the prosencephalon early in embryogenesis and from the telencephalon at later stages of development [1,2]. Like the brain, retinal neurons are terminally differentiated, and post-mitotic cells must survive for as long as the organism does. The multiple visual processes occurring in the vertebrate eye require the production and consumption of huge amounts of energy. It is not surprising that the oxygen consumption of the mammalian retina is higher than that of any part of the adult brain or of other tissues [3,4]. At the base of the outer segment of the photoreceptor, stacks of flat disks are generated daily, whereas disks at the tip are shed and phagocytosed by the adjacent retinal pigment epithelium (RPE) cells [5]. Both processes entail high levels of biosynthetic activity, involving a large number of metabolites. Thus, both RPE and photoreceptor cells consume large amounts of ATP produced by oxidative phosphorylation linked to the mitochondrial electron transport chain.

Paradoxically, while light and oxygen are essential for vision, high levels of oxygen consumption create a stressful environment for neurons. Indeed, metabolic byproducts, primarily reactive oxygen species (ROS), constantly attack neuroretinal genomic and mitochondrial DNA [6,7]. ROS are involved in visible light-induced retinal degeneration [6,8]. Oxidative damage is also implicated in several ocular diseases including inherited retinal dystrophies [9], age-related macular degenerations [10], cataracts, and overexposure to sunlight [11,12]. Oxidative damage accumulates throughout life, contributing to the aging process [13]. The retina is a typical tissue, displaying frequent oxidative damage, including DNA damage; this causes the loss of retinal cells, which is particularly marked during aging [14,15]. As with all other neurons of the CNS, retinal cells are irreplaceable and must maintain the cellular integrity throughout the whole lifespan. These cells therefore require stringent defense mechanisms against ROS-induced damage to ensure their longevity.

Oxidative stress causes many types of DNA damage, such as oxidative DNA damage, single-strand breaks, or double-strand breaks (DSBs) [16]. DNA DSBs are potentially lethal to the cell and therefore must be rapidly recognized and repaired to avoid subsequent genetic damage and cell death or cancerous transformation [17]. Multiprotein complexes involved in DNA maintenance as well as repair also function as sensors of DNA damage. Ataxia-telangiectasia mutated (ATM), ataxia-telangiectasia and Rad3-related (ATR), and DNA-dependent protein kinase catalytic subunit (DNA-PKcs) are members of the phosphoinositide 3-kinase related kinases (PIKKs) family [18,19]. They are activated at very early stages of the DNA damage response and are essential for transducing DNA damage signals to checkpoint control proteins [20]. The ATM protein is a major activator in the cellular response to DSBs, after initial DSB processing by the Mre11/Rad50/Nbs1 (MRN) complex [21]. The ATM interaction domain of the NBS1 C**-**terminus is required to recruit ATM to sites of DNA damage [22-24]. ATM is rapidly activated in a process of autophosphorylation and conversion of inert ATM homodimers or multimers to active monomers [25]. ATM phosphorylates several downstream targets, including p53, murine double minute 2 (MDM2), checkpoint kinase 1 (Chk1), checkpoint kinase 2 (Chk2), breast cancer 1 (BRCA1), Fanconi anemia complementation group 2 (FANCD2), and Nibrin (NBS1). These target proteins are involved in cell cycle checkpoints, stress response, and DSB repair or cell death by apoptosis, and possibly in sensing oxidative stress as well as damage [19,26-29]. ATM is also activated in response to changes in chromatin structure caused by DNA damage [30]. After its rapid activation by autophosphorylation, ATM mediates cellular responses that lead to either repair and cell survival or apoptosis [31].

Loss of ATM function in human and mouse cells causes defects in molecular pathways that are normally activated by DNA DSBs [32]. Mutations in the *ATM* gene lead to ataxia telangiectasia (A-T), an autosomal recessive disorder characterized by progressive ataxia, conjunctival telangiectasias, and other defects including immunodeficiency, cancer susceptibility, chromosomal instability and sensitivity to ionizing radiation (IR) [33,34]. ATM is expressed in both embryonic and adult murine cerebral tissues [35-37]. It is predominantly found in the cell nucleus of most proliferating cells, with smaller amounts of ATM detected in the cytoplasm of these cells [38,39].

ATM is activated in response to increased levels of endogenous as well as induced oxidative DNA damage that includes, but is not necessarily limited to DSBs [26,40]. *Atm*-deficient mice spontaneously develop increased levels of oxidative damage and display mutation profiles suggestive of oxidative stress [41,42]. Elevated ROS levels in *Atm*-deficient mice are particularly marked in Purkinje cells [43]. Oxidative stress is linked to several human diseases, especially cancer and neurologic disorders [14,44], and thus may be involved in the pathogenesis of A-T [31].

Although ocular tissues, especially the retina, are exposed to extremely high levels of ROS, and despite the increased sensitivity of *Atm*-deficient mice to ROS-induced DNA damage, retinal neurodegeneration has not been reported in patients affected by A-T. Most studies aimed at elucidating the DSB response have been performed in proliferating cell lines. Nevertheless, it was recently shown that an intact DNA damage response is essential for normal development, organization, and maintenance of the nervous system [45]. The pro-apoptotic ATM response to IR in the developing mouse CNS suggests that ATM functions at a developmental survival checkpoint, serving to eliminate neurons with excessive DNA damage [46,47]. The selective nature of IR-induced ATM-dependent apoptosis is p53-dependent and related to the differentiation status of cells [48,49]. Indeed, ATM-dependent apoptosis may be important for the development and maintenance of the nervous system.

Recent studies have reported that the role of ATM in the DNA damage response in the CNS—including the retina—is conserved between several species. In the zebrafish, surveillance of genome integrity is ATM-dependent. Indeed, a zebrafish mutant, *pinball eye* (*piy*), in which almost all retinal neurons undergo apoptosis during differentiation, has been recently isolated. A missense mutation in a small subunit of DNA primase (Prim1) underlies this mutant phenotype. This mutation does not affect cell proliferation but specifically induces extensive retinal neuronal apoptosis by activation of the ATM-Chk2-p53 pathway [50]. Similarly, ATM knockdown in *Drosophila* eyes causes progressive degeneration of photoreceptors in the absence of exogenously induced DNA damage [51]. In this model, post-mitotic neurons re-entered in the cell cycle, indicating that ATM is involved in a cell-cycle checkpoint and prevents post-mitotic neurons from undergoing degeneration.

The rare diseases A-T and Nijmegen breakage syndrome (NBS)-with mutations in the *Nbs1* gene encoding the Nibrin protein-share a variety of phenotypic abnormalities, such as spino-cerebellar ataxia, chromosomal instability, radiation sensitivity, and defects in cell-cycle checkpoints in response to IR. The similarity between A-T and NBS may be a result of biochemical links between ATM and NBS1 in the DNA damage response and cell cycle regulatory pathways [52-55]. Indeed, the targeted disruption of murine *Nbs1* gene in the CNS shows a neurologic phenotype in these animals, characterized by the arrested proliferation of granule cell progenitors and apoptosis of post-mitotic neurons in the cerebellum; this leads to severe ataxia and microcephaly, typical clinical features of NBS in humans [56]. These mice also present microphthalmia due to reduced proliferation of the lens epithelial cells and early onset cataracts due to altered lens fiber cell differentiation [57]. *Nbs1* disruption also leads to a dramatic impairment in the development of the eye and optic nerve, with severe dysfunction of the photoreceptors [45]. This animal model recapitulates many clinical aspects of the neurologic phenotype in A-T patients, whereas *Atm*-deficient mice do not exhibit this neurologic phenotype. A simple explanation for the phenotypic differences between *Atm*-deficient mice and A-T patients could be that generation of the A-T cerebellar phenotype in the mouse requires a greater suppression of the DNA damage response than that achieved by deletion of the *Atm* gene [58].

A recent study suggested that the formation of retinal and choroidal vascular abnormalities (idiopathic perifoveal telangiectasia) observed in patients of European-American descent is associated with *ATM* missense variants [59], consistent with findings of an earlier study [60]. These findings further support the notion that neuronal degeneration is caused by a defect in the DNA damage response in both human A-T and animal models.

These studies demonstrate a fundamental role for ATM both in the development and maintenance of the eye. However, no previous systematic study has yet determined the precise localization of both *Atm* mRNA and protein in the adult murine eye. Thus, we performed such a study to better understand both the physiologic role of ATM in the adult murine eye and the pathological features of A-T, which seem to have a broader spectrum of ocular manifestations than previously thought.

In this study, we determined the distribution pattern of *Atm* mRNA and the localization of ATM protein in the adult mouse eye. We showed that ATM protein was essentially localized in the nucleus of retinal cells and non-neuronal cells of the mouse adult eye. High levels of the activated phosphorylated form of ATM (ATMp) were detected in the nucleus and the cytoplasm of most ocular cell types. Additionally, immunoreactivity was detected in the same cells for both ATM and ATMp; however, ATMp cellular localization in photoreceptor cells differed from that detected in other retinal cells. In mouse photoreceptors, ATM immunostaining seemed to be exclusively in the nuclei, whereas ATMp immunostaining was confined to the cytoplasmic compartment. The specific patterns of ATM and ATMp immunoreactivities appeared to be the same in the mouse cerebellar granule cells as those observed in mouse photoreceptor cells. This may be indicative of a particular susceptibility of these cells to oxidative damage. Our findings suggest that ATM and its phosphorylated form have common and redundant functions as well as distinct and specific functions in the maintenance of the integrity of ocular cells.

## Methods

### Animals

All animals were handled in strict accordance with the ARVO Statement for Use of Animals in Ophthalmic and Vision Research. C57BL/6J mice (Charles River, L'Arbresle, France) were maintained on a 12 h:12 h light-dark cycle at a room temperature of 21 °C.

### RT–PCR and PCR

*Atm* mRNA in the cerebellum, neuroretina, RPE, ciliary body, and lens from 2-month-old adult mice were examined by RT–PCR. Total RNA was extracted from each sample of each mouse ocular compartment and from each mouse cerebellum by TRIzol method, according to the manufacturer’s recommendations (Invitrogen, Cergy-Pontoise, France). Next, 1 μg aliquots of total RNA were reverse transcribed with reverse transcriptase (SuperScript II, Invitrogen) and oligo dT primer, according to the manufacturer’s instructions.

PCR conditions such as MgCl_2_ concentrations, annealing temperatures and number of cycles were optimized for *Atm* and *cyclophilin A* genes to perform PCR in the linear part of amplification [61]. We determined the appropriate number of cycles so that the amplification product was clearly visible on an agarose gel and could be quantified, but also so that amplification was in the exponential range and had not yet reached a plateau. The optimal number of cycles has to be in the same range for the specific RNA of interest (i.e., *Atm*) and the control, in this case *cyclophilin A*, to be measured by the comparison of the respective intensities of their distinct PCR amplified products on the same gel.

PCR reactions were performed in 25 µl of reaction mixture containing 2 µl cDNA, 0.2 µM of each *Atm* primer, and 0.04 µM of each *cyclophilin A* primer, 0.2 mM dNTP, 1.5 mM MgCl_2_, 2.5 µl 10X PCR buffer, and 0.05 U Taq DNA polymerase (Invitrogen). Semiquantitative PCR was performed with a denaturing step of 94 °C for 4 min, followed by 30 cycles (optimal cycling) of 94 °C for 30 s, 55 °C for 30 s, 72 °C for 1 min, then a final extension of 7 min at 72 °C. *Atm* primers (forward 5′- ATC CCT TGT GTG TTC TCT G −3′ and reverse 5′- CGC CTC TGC TGT CGT GTA T −3′) and *cyclophilin A* primers (forward 5′-TGG TCA ACC CCA CCG TGT TCT TCG-3′ and reverse 5′-TCC AGC ATT TGC CAT GGA CAA GA-3′) amplified 472 bp and 311 bp products, respectively.

PCR products were separated on 1% agarose gels and visualized by ethidium bromide staining under UV light. Each RT–PCR reaction was done in triplicate with three different samples for each microdissected tissue extract. The image of the ethidium bromide-stained gel was digitalized, using a digital video image analyzer ImagerTM (Appligene, Illkirch, France), and analyzed using ImageJ 1.40 g software.

We systematically verified that all the PCR reactions were performed during their exponential phase, also called the linear phase, and, thus, that we had not yet reached the plateau phase.

### Statistical analysis

PCR signals were normalized as a function of the *cyclophilin A* gene expression, which reflects the initial amount of RNA, by making the ratio between the signal obtained for this *Atm* gene and that obtained on the control standard curve for the same quantity of *cyclophilin A*. Optical densities of the PCR bands were measured using ImageJ 1.40 g software. Statistical analysis was performed using ANOVA (ANOVA, Statview Software program, version 5) to detect significant intergroup differences. Values are expressed as mean±SEM, and a p<0.001 was considered statistically significant. Comparisons were made between retina and other ocular tissues using a Student–Newman–Keuls test.

### Nonradioactive in situ hybridization

Eyes from adult C57BL/6J mice were fixed by incubation in 4% paraformaldehyde in 0.1 M phosphate buffer (pH 7.4) at 4 °C, embedded in paraffin, and cut into 5 μm sections. The *Atm* PCR product was inserted into the pCRII-TOPO TA Cloning vector (Invitrogen). Products were checked by sequence analysis. Digoxigenin (DIG)-labeled riboprobes were synthesized by in vitro transcription using the standard DIG-labeling reaction protocol from Promega (Charbonnieres, France). T7 and SP6 RNA polymerase were used for the sense and antisense riboprobes, respectively.

Tissues were deparaffinized by incubation in xylene and rehydrated through a graded series of alcohol solutions. Next, 150 ng RNA probes were diluted in mRNA HIS Solution (Dako, Trappes, France) and incubated with sections at 60 °C overnight in a humidified chamber. After washing in Stringent Wash Concentrate 50X (Dako), labeling was detected by incubation with 1:500 alkaline phosphatase-coupled anti-DIG antibody in antibody diluent (Dako) for 1 h at room temperature. *Atm* mRNA hybridization signals were developed with the BCIP/NBT Substrate System (10–15 min at room temperature; Dako).

### Immunostaining

#### Immunohistochemistry

Rabbit polyclonal anti-ATM (H-300), used at a dilution of 1:300, and goat polyclonal anti-recoverin (C-15), used at a 1:200 dilution, antibodies were purchased from Santa Cruz Biotechnology (Tebu, Le Perray, France). We used three antibodies against phosphorylated ATM (pS1987 in mice that correspond to pS1981 in human) that display the same immunolabeling: rabbit polyclonal antibody pS1987 (ab2888), used at 1:50 dilution, was purchased from Abcam (Paris, France), mouse monoclonal antibody pS1987 (200–301–500), used at a 1:200 dilution, was purchased from Rockland (Gilbertsville, PA) and rabbit polyclonal antibody pS1987 (11122), used at a 1:200 dilution was purchased from Signalway antibody (Pearland, TX). Mouse monoclonal anti-γH2AX antibody (ser139, clone JBW301), used at a 1:500 dilution and mouse monoclonal anti-8-oxoguanine antibody (N45.1), used at a 1:40 dilution, were purchased from Upstate (Temecula, CA) and Gentaur (Brussels, Belgium) respectively.

Paraffin sections were deparaffinized in Xylene and boiled in a microwave oven for two periods of 10 min in a citrate buffer composed of 0.01 M citric acid monohydrate, pH 6.0. Sections were incubated with primary antibody in antibody diluent (Dako) in a humidified chamber overnight at 4 °C. The ChemMate peroxidase/DAB Rabbit/Mouse detection kit (Dako) was used to detect bound antibodies, using the reaction protocol. Sections were washed in 1X PBS (137 mM NaCl; 2.7 mM KCl; 4.3 mM Na_2_HPO_4_; 1.47 mM KH_2_PO_4_; pH 7.4) between each step. After DAB staining, sections were counterstained with methyl green solution and mounted with Eukitt (PolyLabo, Strasbourg, France). For immunohistochemical staining of 8-oxoguanine (8-oxo-dG) in DNA, ribonucleic acids were removed from sections with 20 µg/ml Rnase solution (Invitrogen) and tissue DNA was denatured in 2N HCl for 5 min before incubation with primary antibody. The specificity of the antibody raised against the 8-oxo-dG was tested. Experiments were performed not only in normal adult retina but also in normal and Ogg1 knockout mice livers (data not shown). This modified base is known to be accumulated in the liver of *Ogg1^−/−^* mice as compared to the normal liver of control mice [62]. These experiments provided us with additional positive controls for further validation of the specificity of the antibody raised against 8-oxo-dG.

#### Immunohistofluorescence

Sections were incubated with primary antibody in antibody diluent (Dako) in a humidified chamber overnight at 4 °C. Sections were washed and incubated with a 1:200 dilution of secondary Alexa Fluor 488 donkey anti-rabbit (A21206), Alexa Fluor 546 donkey anti-goat (A11056), or Alexa Fluor 488 goat anti-mouse (A11017) antibodies (Molecular Probes, Invitrogen) in antibody diluent (Dako) in a humidified chamber for 2 h at room temperature in the dark. Washed sections were mounted in Fluorescent Mounting Medium (Dako). Sections were counterstained with 1:1,000 propidium iodide (PI, Sigma-Aldrich, Saint-Quentin Fallavier, France) or 1:1,000 TO-PRO-3 (Invitrogen).

### Confocal microscopy and colocalization

Optically sectioned images were acquired by confocal laser scanning microscopy using a Leica SP5 confocal microscope and analyzed using Leica Microsystems LAS AF software (1.8.2). Two labels colocalized if they were too close in the tissue to be resolved optically. The extent of colocalization of two labels was measured using the “Colocalization” module of Imaris software (6.1.2; Bitplane, Zurich, Switerland), described previously [63]. Briefly, this program analyzes images of confocal sections acquired in two channels. Each confocal section consists of an array of square elements called pixels. A voxel is defined as a prism in which the base is the pixel and the height is the thickness of the confocal section. Imaris colocalization analyzes the entire confocal image by measuring the intensity of each label in each voxel. An intensity threshold (on a scale of 0–255 for pixel intensity) for each of the two labels is determined. In this study, we used a threshold of 30 (on a scale of 0–255) for each label. Voxel intensity values above this threshold were considered to be above background intensity. A voxel was defined as displaying colocalization when the intensity of both labels was above their respective thresholds. The percentage of material colocalized was based on the number of voxels displaying colocalization and the intensities (material) of the two labels in each voxel. The Pearson coefficient measures the correlation between the values of intensity for the two labels in voxels showing colocalization. The Pearson coefficient lies between +1 and −1, with positive values indicating a direct correlation and values near 0 indicating no correlation.

### Western blot

Total protein lysates were prepared in a Potter-homogenizer with cold buffer composed of 150 mM Nacl, 10 mM Tris HCL, 1 mM EDTA, 1 mM EGTA, 2 mM NaOV, 190 mM NaF, 2 mM PMSF, 1% Triton X100, 0.5% NP-40, and protease inhibitor cocktail (Sigma-Aldrich). Protein concentration was determined using a Lowry protein assay kit (BioRad DC protein assay, Ivry-sur-Seine, France). Proteins were separated on 7% polyacrylamide gels. Each lane was loaded with 100 µg of protein extract. Separated proteins were transferred onto a nitrocellulose membrane and blocked with 5% skim milk for 1 h. Blots were incubated with either the monoclonal antibody against Mat-3 (generously provided by Professor Yosef Shiloh from Tel Aviv University, Tel Aviv, Israel) diluted 1:500 with 1% BSA in PBS-Tween for 3 h at room temperature or with the monoclonal antibody against ATMp (200–301–500) purchased from Rockland (Gilbertsville, PA) and diluted 1:500 with 1% BSA in PBS-Tween overnight. In parallel, membranes were incubated with 1:1,000 goat anti-β-Actin antibody (Tebu) for 2 h at room temperature. Proteins were detected by chemiluminescence (ECL, PerkinElmer Life, Courtaboeuf, France) on light-sensitive film (Biomax Light Kodak; Sigma-Aldrich). *Atm^−/−^* mouse tissues were used as controls for nonspecific binding.

## Results

### Expression and localization of DNA lesions in adult mouse ocular tissues

High amounts of 8-oxo-dG (a major oxidized form of guanine) are produced, both in genomic and mitochondrial DNA, by ROS in the retina, exposed to normal conditions of illumination and functioning in basal physiologic conditions. To evaluate the extent of oxidative damage of cellular DNA under basal conditions, we examined immunohistochemically the retinal and cerebellar distribution of 8-oxo-dG in the C57BL/6J mouse strain, using an anti-8-oxo-dG monoclonal antibody.

**Figure 1:**
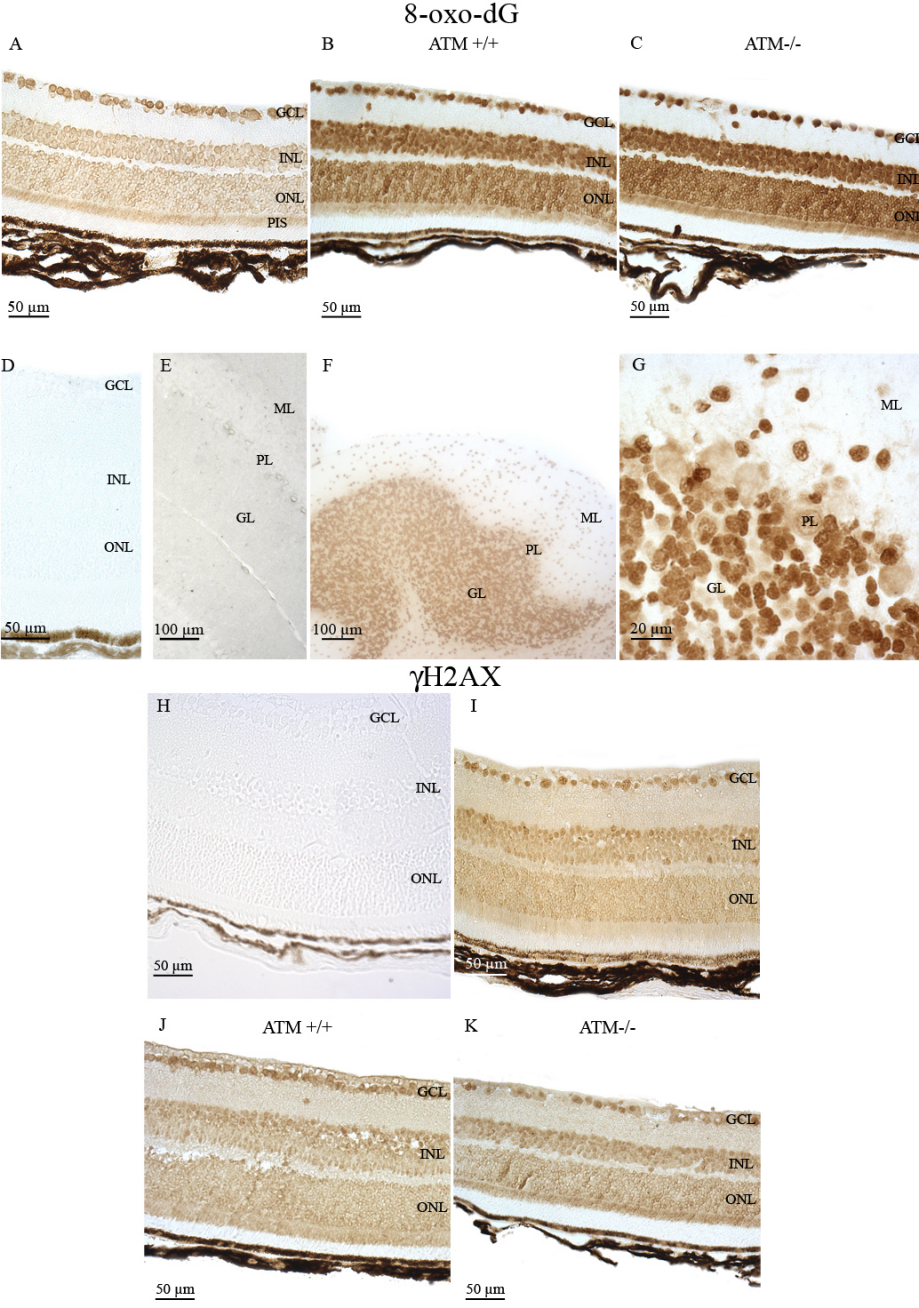
Immunohistochemical localization of 8-oxoguanine and γH2AX. Specific mouse 8-oxoguanine (8-oxo-dG) immunostaining was detected in adult mouse ocular and cerebellar tissue sections. The cellular distribution of 8-oxo-dG immunostaining was observed in retinal cells of C57BL/6J (**A**), *Atm*+/+ (**B**), and *Atm*−/− mice (**C**). No specific retinal (**D**) or cerebellar (**E**) signal could be detected in control experiments. The 8-oxo-dG immunostaining was also observed in cerebellar cells (**F**). A high magnification of 8-oxo-dG immunostaining in the Purkinje cell layer (PL; **G**) is shown. Specific mouse γH2AX immunostaining was detected in adult mouse ocular tissue sections. No specific immunoreactivity was detected in control experiments (**H**). γH2AX immunostaining was observed in C57BL/6J (**I**) *Atm*+/+ (**J**) and *Atm*−/− mouse retina (**K**). Abbreviations: ganglion cell layer (GCL); granular cell layer (GL), inner nuclear layer (INL); molecular cell layer (ML), outer nuclear layer (ONL), and photoreceptor inner segment (PIS).

Strong immunoreactivity for 8-oxo-dG was easily detected in all retinal cell layers and in photoreceptor inner segments (PIS; Figure 1A). The PIS layer is highly enriched in mitochondria and peroxysomes. We further expanded our observations concerning the detection of DNA lesions by studying the 8-oxo-dG immunoreactivity in the control (*Atm^+/+^*) mouse strain and the *Atm*-deficient mice (*Atm^−/−^*). These strains have an sv129 genetic background. Both C57Bl/6J and sv129 mouse strains displayed the same 8-oxo-dG retinal immunostaining pattern (Figure 1B,C). Moreover, we also observed in cerebellar neurons an intense 8-oxo-dG immunoreactivity, mostly in the nuclei of the granule (GL) but also in the molecular layer (ML) of the cerebellar cortex (Figure 1F,G). Faint 8-oxo-dG immunolabeling was observed in the nuclei of the Purkinje cell layer (PL).

The physiologic oxidative stress existing in photoreceptor cells is very likely the source of DSBs [16] which, even if they might not be the major DNA lesions occurring in these neural sensory cells, constitute certainly an important percentage of the total DNA lesions affecting the mitochondrial and genomic DNA of these retinal cells specialized in phototransduction. We detected γH2AX, largely considered as a reliable marker of DSBs lesions, in the normal mouse retina (C57Bl/6J and *Atm^+/+^* mouse), in the *Atm* knockout mice retina (Figure 1 I-K), and in cerebellar neuronal cells of all mouse strains studied (cerebellar data not shown). The detection of this strong immunoreactivity in all retinal cells and especially in photoreceptor cells also strengthens the hypothesis suggesting that basal functional conditions in these cells are associated with a high oxidative stress linked to a huge production of ROS. This production is at the origin of DNA damage including DSBs both in genomic and mitochondrial DNA, since γH2AX immunoreactivity is strongly observed both in photoreceptors nuclei and PIS.

### Expression and localization of *Atm* mRNA in adult mouse ocular tissues

**Figure 2:**
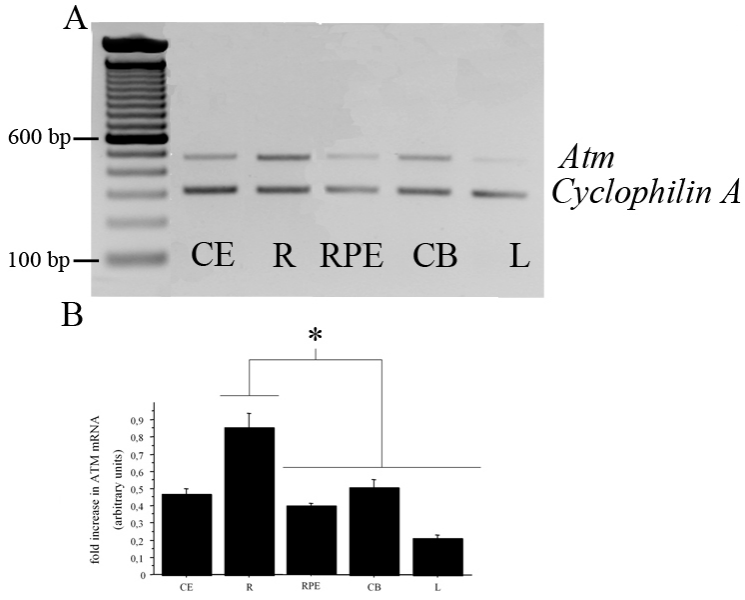
*Atm* mRNA in adult mouse cerebellum and retina, with associated densitometric analysis of the PCR bands. Semiquantitative RT–PCR was used to determine the relative amounts of *Atm* and *cyclophilin A* mRNAs in C57BL/6J mouse cerebellum (CE), neuroretina (R), retinal pigment epithelium (RPE), ciliary body (CB), and lens (L) at P60. **A**: The 472-bp and the 311-bp bands correspond to *Atm* and *cyclophilin A* PCR products, respectively. The *cyclophilin A* cDNA band obtained by RT–PCR was used as an internal control. **B**: Results represent the ATM cDNA relative levels calculated as the ratios of the intensities of the *Atm* and *cyclophilin A* bands (n=3). Values are expressed as mean±SEM, and ANOVA was performed to determine the difference between tissues. Asterisk (*) indicates p<0.001.

We investigated *Atm* mRNA levels in four distinct murine ocular tissular compartments and in the cerebellum by semiquantitative RT–PCR (Figure 2). As expected, a 472 bp band, corresponding to *Atm* mRNA, was observed in all mouse samples tested (cerebellum, neuroretina, RPE, ciliary body, and lens; Figure 2A). *Cyclophilin A* cDNA band intensity (used as an internal control) varied less than 5% between each lane of electrophoresis migration in all our experiments and was used to normalize variations in *Atm* expression. *Atm* mRNA was detected in each mouse ocular tissue sample examined; bands of the same size were observed in both ocular and cerebellar tissues. *Atm* mRNA levels were significantly higher in the neuroretina than in any other ocular tissues (Figure 2B).

**Figure 3:**
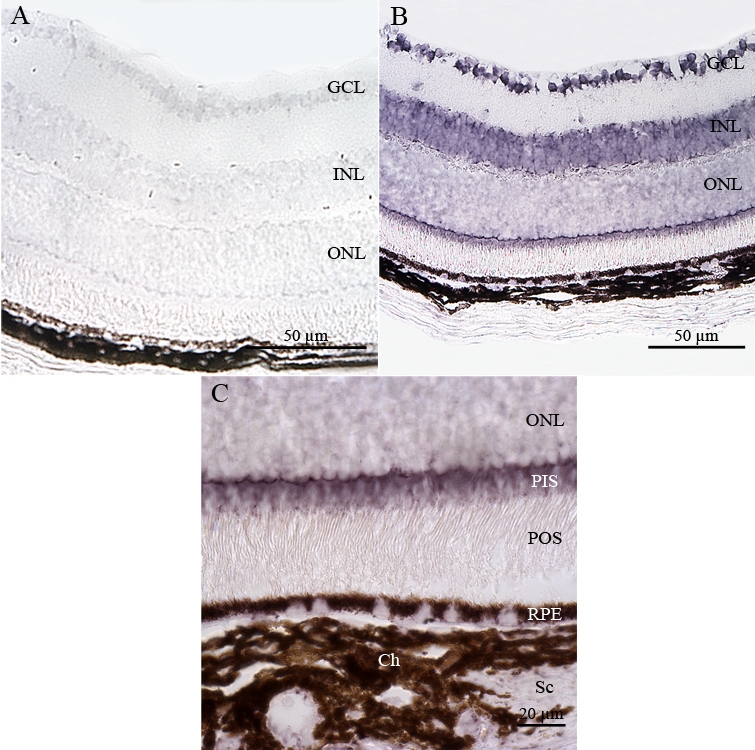
Distribution of *Atm* mRNA in adult mouse retina. *Atm* mRNA was reproducibly detected in adult mouse retinas by in situ hybridization using specific antisense digoxigenin (DIG)-labeled riboprobe. No specific hybridization signal was detected with the sense probe (**A**). **B:** Labeled *Atm* mRNA appeared as dark blue or purple precipitates in all nuclear layers of the retina. **C:** A high magnification of the outer portion of the neuroretina is shown. Abbrevations: choroid cells (Ch), ganglion cell layer (GCL), inner nuclear layer (INL); outer nuclear layer (ONL); photoreceptor inner segment (PIS), photoreceptor outer segment (POS), retinal pigment epithelium (RPE), and scleral cells (Sc).

To determine the cellular sites of *Atm* mRNA expression in the adult mouse ocular compartments, we analyzed paraffin-embedded tissue sections of adult C57BL/6J mouse eyes (P60) by nonradioactive in situ hybridization using DIG-labeled sense and antisense riboprobes [64]. As expected, no specific labeling was detected in any ocular tissue sections hybridized with the *Atm* sense probe (Figure 3A). *Atm* mRNA was detected in all neuroretinal nuclear layers such as ganglion cell layer (GCL), inner and outer nuclear layer (INL and ONL) and in PIS (Figure 3B). No significant *Atm* hybridization signal was detected in the photoreceptor outer segments (POS) as well as the inner and outer plexiform layers (IPL and OPL). Expression of *Atm* mRNA seemed higher in the PIS, INL, and GCL than in the ONL. *Atm* mRNA was also expressed in the RPE cell layer, which is adjacent to the neural retina and is also derived from the CNS (outer layer of the optic cup; Figure 3C). The systematic bioinformatic analysis of ESTs databases and tissue specific cDNA profiling databases allowed us to uncover very interesting data obtained independantly by other teams about *Atm* gene expression. The most interesting data are released in the BioGPS website and correspond to an *Atm* gene expression/activity chart. They are in complete adequation with the results presented in this report. This chart confirms the significant levels of expression and activity of *Atm* detected in cornea, iris, ciliary body, RPE, retina, and eyecup. This chart indicates strikingly that the ratios of *Atm* gene expression in most compartments of the eye and especially in the retina are comparable to that detected in the cerebellum.

**Figure 4:**
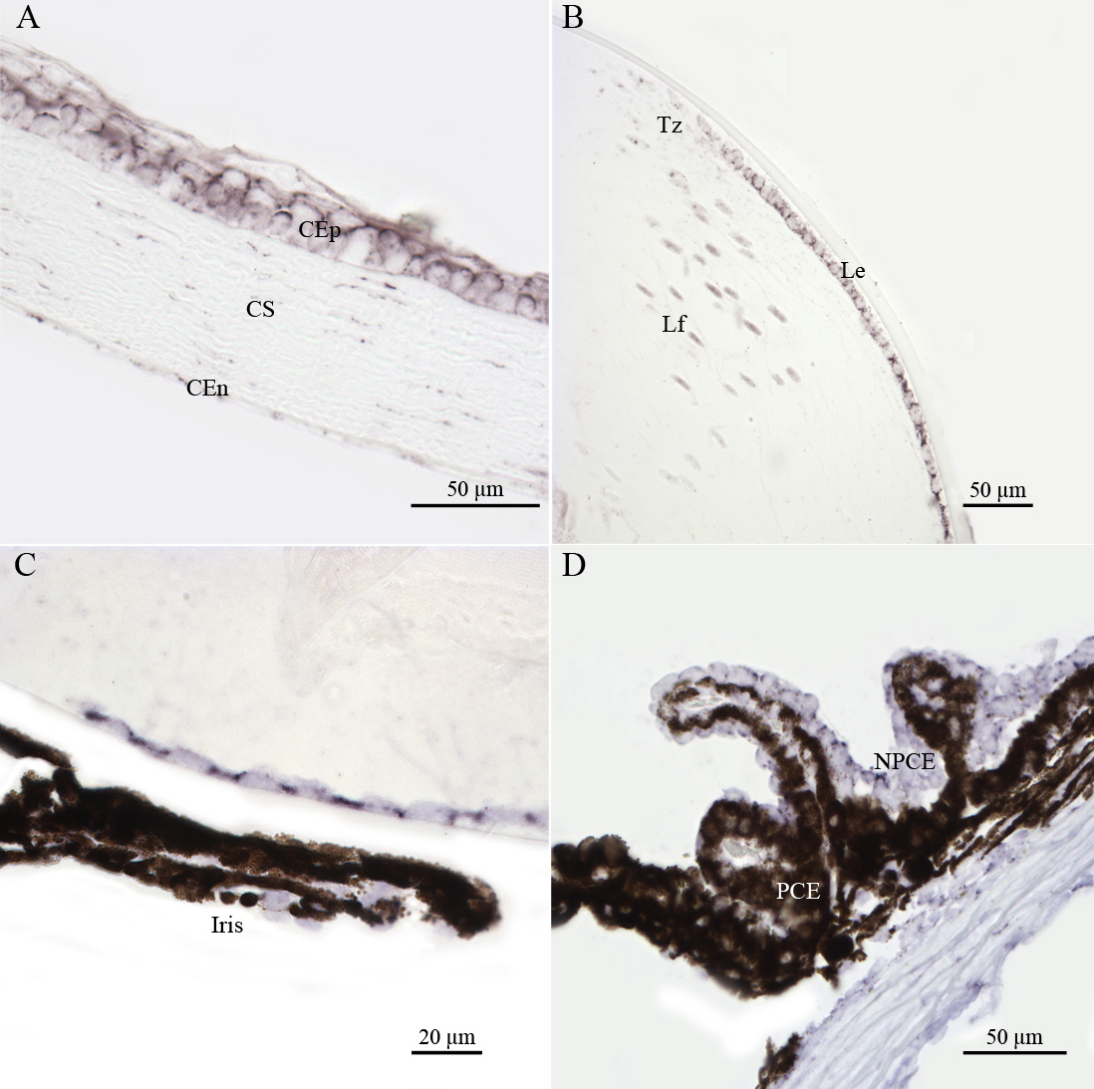
Distribution of *Atm* mRNA in nonretinal compartments of adult mouse ocular tissue sections. In situ hybridization signals of *Atm* mRNA were detected, using a specific digoxigenin labeled probe, in the corneal epithelial (CEp), corneal stromal (CS), and corneal endothelial (CEn) cells (**A**), in the lens epithélium (Le), transitional zone (Tz), and lens fibers (Lf; **B**), in iridal cells (**C**), and in pigmentary ciliary epithelium (PCE) and nonpigmentary ciliary epithelium (NPCE) cells (**D**). These results demonstrate that Atm gene is transcribed both in dividing and non dividing nonretinal cells of the adult mouse eye.

Non-neuronal ocular tissues, including choroidal (Ch) and scleral (Sc) cells, also produced *Atm* transcripts (Figure 3C). We detected significant levels of *Atm* mRNA in the corneal epithelial cells (CEp), corneal stromal keratocytes (CS) and corneal endothelial cells (CEn) (Figure 4A). In the lens, *Atm* mRNA was readily detected in the lens epithelium (Le), the transitional zone (Tz), and in elongating fiber cells (Lf), in which the nucleus was still visible (Figure 4B). Iridal cells also showed significant *Atm* mRNA hybridization signals (Figure 4C). The pigmented ciliary epithelial (PCE) and nonpigmented ciliary epithelial (NPCE) cells of the ciliary body both displayed *Atm* mRNA hybridization signals (Figure 4D).

**Figure 5:**
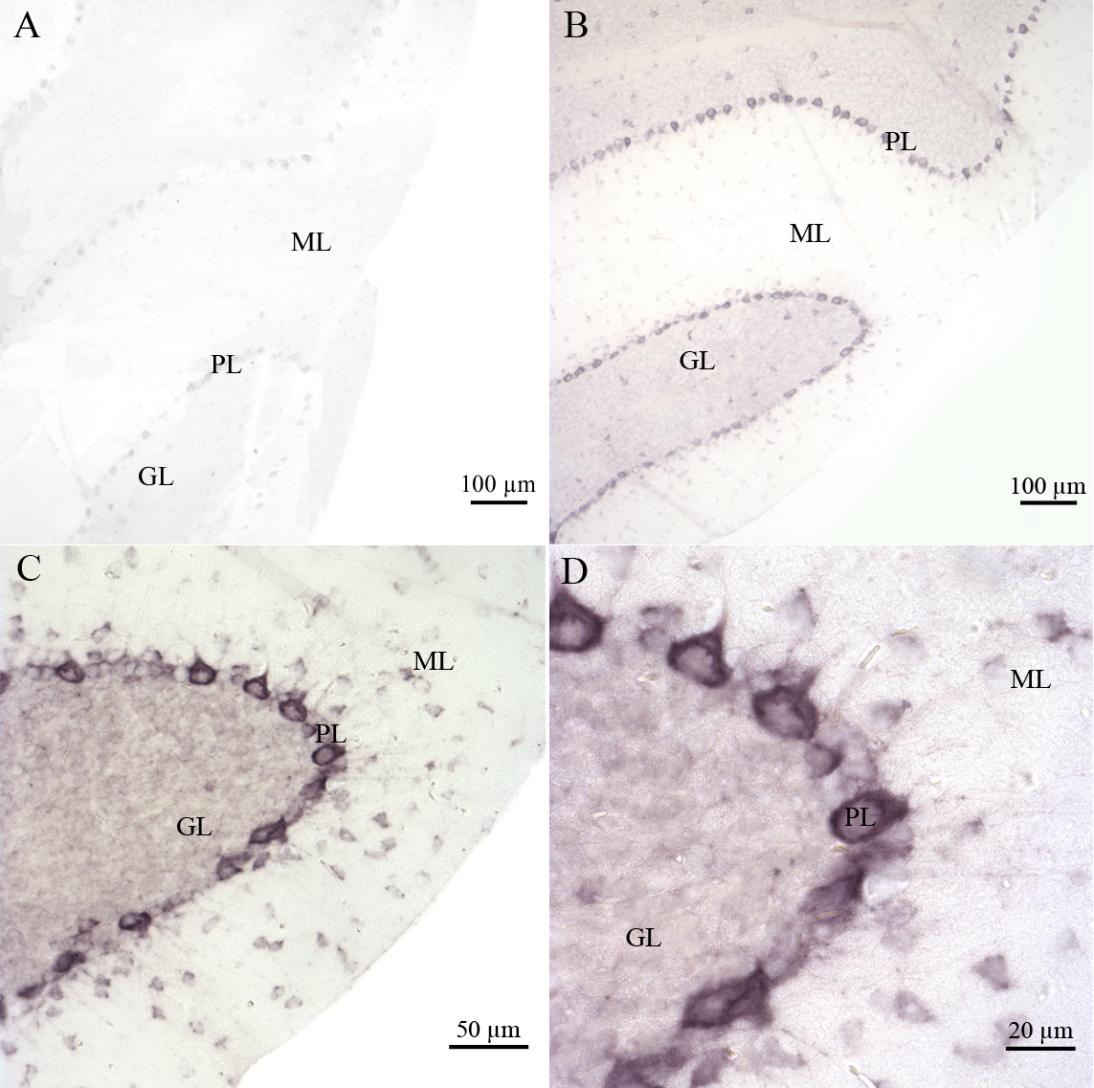
Distribution of *Atm* mRNA in the adult mouse cerebellum. No specific signal was detected with the sense probe (**A**). Labeled *Atm* mRNA was detected in adult mouse cerebellum tissue sections by in situ hybridization using specific digoxigenin-labeled antisense riboprobe (**B**). A high magnification of *Atm* hybridization signals in the granular cell (GL), molecular cell (ML), and Purkinje cell (PL) layers is shown (**C-D**).

We also characterized *Atm* expression in normal mouse cerebellum tissue sections. This served as a positive control for our experiments on adult mouse ocular tissues. As expected, no specific labeling was detected in any cerebellar tissue sections hybridized with the *Atm* sense probe (Figure 5A). *Atm* mRNA was mainly detected in the granule GL and ML of the cerebellar cortex (Figure 5B,C). Strong labeling was also detected in the PL (Figure 5D).

### Expression and localization of ATM protein in adult mouse ocular tissues

**Figure 6:**
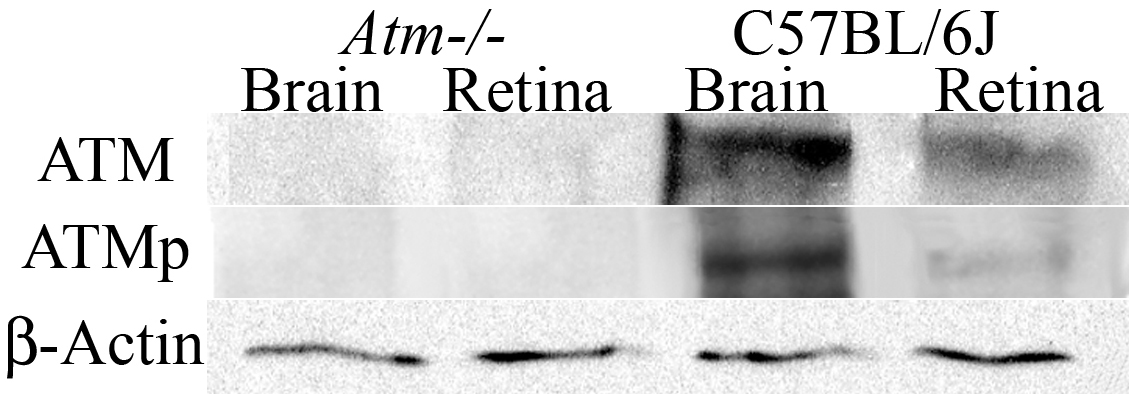
Determination of ATM and ATMp antibodies specificity by western blotting. Western blot analysis of neuroretinas and brains from adult *Atm*-deficient and adult C57BL/6J mice was performed with monoclonal antibodies raised against ATM and ATMp, and with polyclonal antibody β-actin (internal control). Specific bands for ATM (350 kDa), ATMp (350 kDa), and β-actin (35 kDa) were detected by western blot analysis of protein homogenates extracted from different mouse tissues (neuroretina and brain). No band could be detected in protein homogenates from *Atm*-deficient mice tissues blotted with either specific ATM or specific ATMp antibody.

To confirm our findings from the nonradioactive in situ hybridization experiments, we used immunohistochemistry to investigate the presence of unphosphorylated ATM in adult mouse ocular paraffin-embedded tissue sections, especially in the retina. The specificity of the anti- ATM antibody was confirmed in western blots of proteins homogenates from mouse neuroretina or brain. Identical single bands corresponding to a protein of a molecular weight around 350 kDa were reproducibly detected for each tissue extract tested. This band corresponded to the expected size of ATM, reported in previous publications [65]. This band was not detected in *Atm*-deficient mice proteins homogenates (Figure 6). These results demonstrate the specificity of the monoclonal anti-ATM antibody used in our experiments.

**Figure 7:**
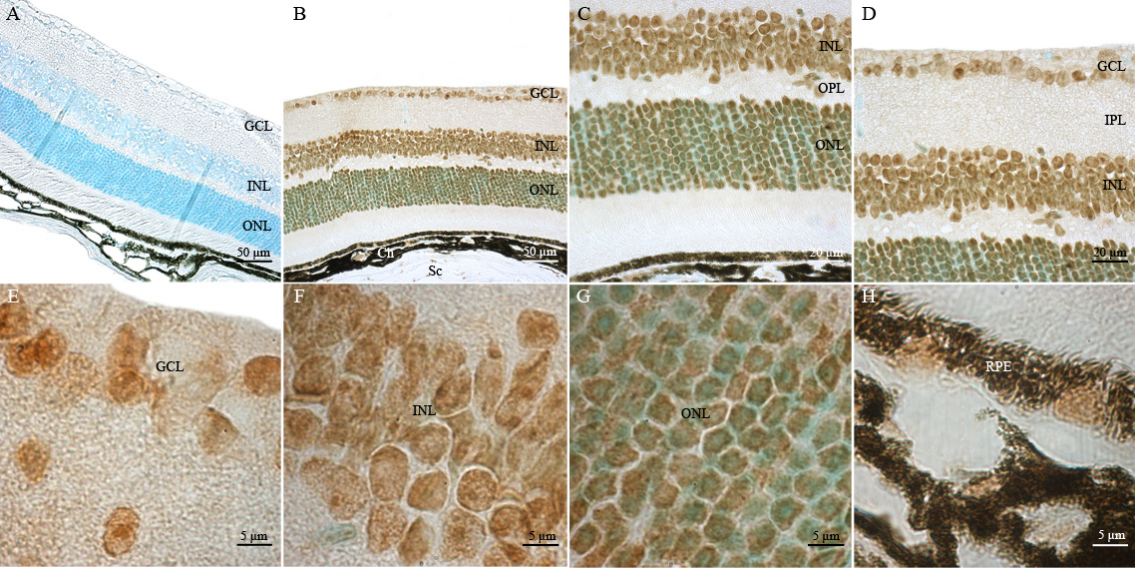
Immunohistochemical localization of ATM in the adult mouse neuroretina. Immunostaining using a polyclonal antibody raised against ATM in neuroretina. No specific signal was detected when the specific anti-ATM primary antibody was omitted (**A**). ATM immunostaining was brown, and sections were counterstained with a methyl green solution. The cellular distribution of ATM immunostaining was observed in retinal cells (**B**). A high magnification of the outer part (**C**) and inner part (**D**) of the neuroretina is shown. A high magnification of the ganglion cell layer (GCL; **E**), inner nuclear layer (INL; **F**), outer nuclear layer (ONL; **G**) and retinal pigment epithelium (RPE; **H**) is shown. Abbreviations: choroid (Ch); inner plexiform layer (IPL), outer plexiform layer (OPL), and scleral cells (Sc).

The presence of ATM protein in eye tissue sections from adult mice was investigated by immunohistochemistry (Figure 7). No ATM immunostaining was observed in the appropriate control experiments (Figure 7A). We detected ATM protein in the nuclei of the ONL, INL, and GCL (Figure 7B-G). ATM immunoreactivity was also observed in the RPE cell layer and in non-retinal cells such as the Ch cells, including uveal and vascular endothelial cells, and Sc cells (Figure 7B,H). Interestingly, ATM immunostaining in the ONL, INL, and GCL appeared to be of similar intensity (Figure 7B-D). In contrast, the intensity of the in situ hybridization signal appeared to be weaker in the ONL than in the INL or GCL (Figure 3B). ATM protein in the ONL might be subject to post-transcriptional regulation; further study is required to confirm this hypothesis. These results are consistent with a previous study suggesting that ATM is post-transcriptionally regulated in proliferating cells such as peripheral blood mononuclear cells [66]. Thus, ATM may be post-transcriptionally regulated in both dividing and post-mitotic cells.

**Figure 8:**
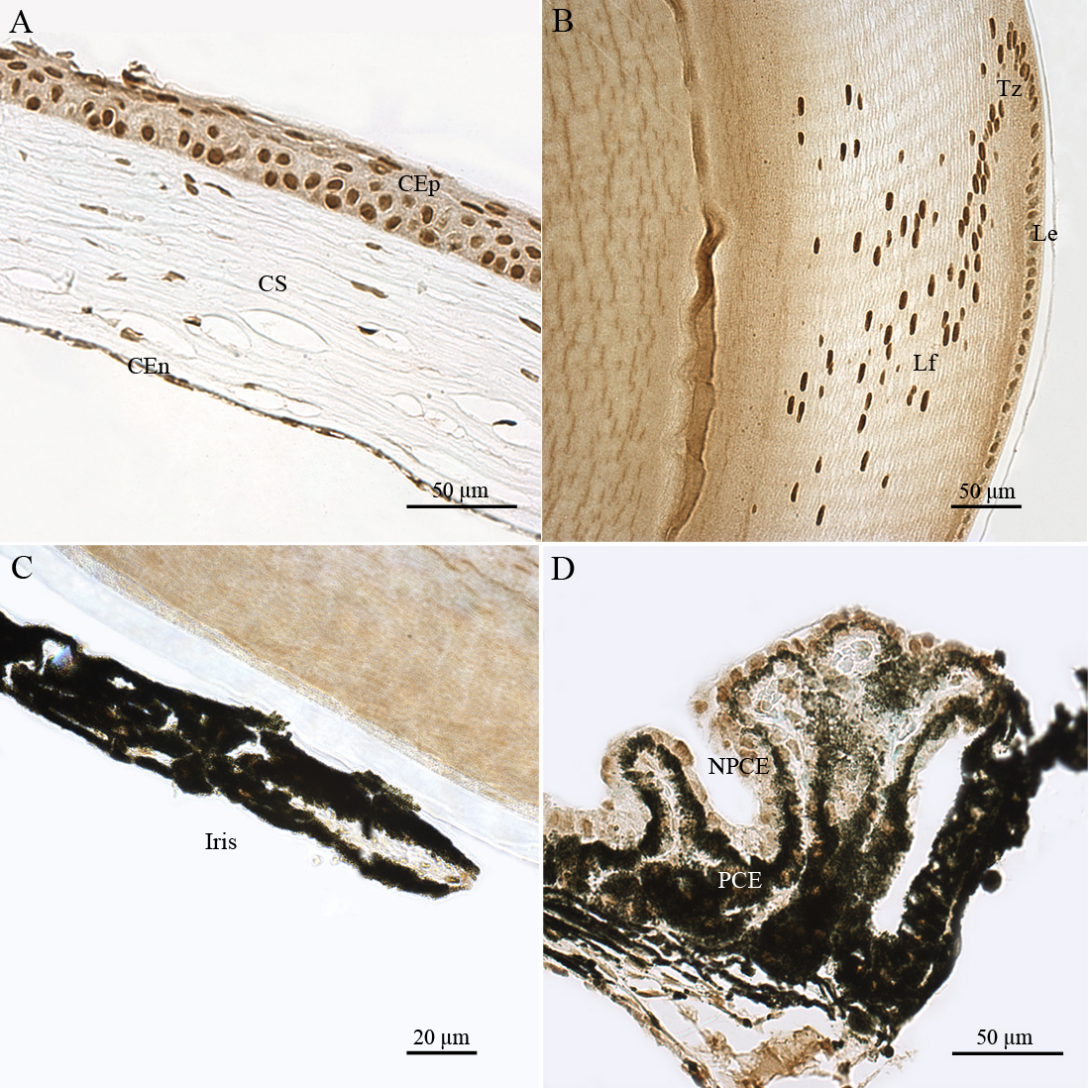
Immunohistochemical localization of ATM in nonretinal cells of adult mouse ocular tissue sections. ATM immunolabeling was observed in corneal epithelial (CEp), corneal stromal (CS), and corneal endothelial (CEn) cells (**A**); in the lens epithelium (Le), transitional zone (Tz) and lens fiber (Lf; **B**); in iridal cells (**C**) and in the pigmentary ciliary epithelium (PCE) and nonpigmentary ciliary epithelium (NPCE) cells (**D**). ATM immunoreactivity is primarily detected in the nucleus of nonretinal ocular cells

ATM immunostaining was also detected in other non-neuronal ocular cells, including the nuclei of CEp, CS, and CEn (Figure 8A). ATM immunolabeling was observed in the nuclei of the Le, the Tz, and the Lf (Figure 8B). ATM was slightly detected in all iridal cells, with signal in both pigmented layers of the iris and iridal uveal cells (Figure 8C). It was also present in the nuclei of ciliary processes. Both the PCE and the NPCE cell layers had marked ATM immunostaining (Figure 8D). All cell types observed in the ciliary body showed significant levels ATM immunoreactivity, including those located at the root of the ciliary body and at the iridocorneal angle.

**Figure 9:**
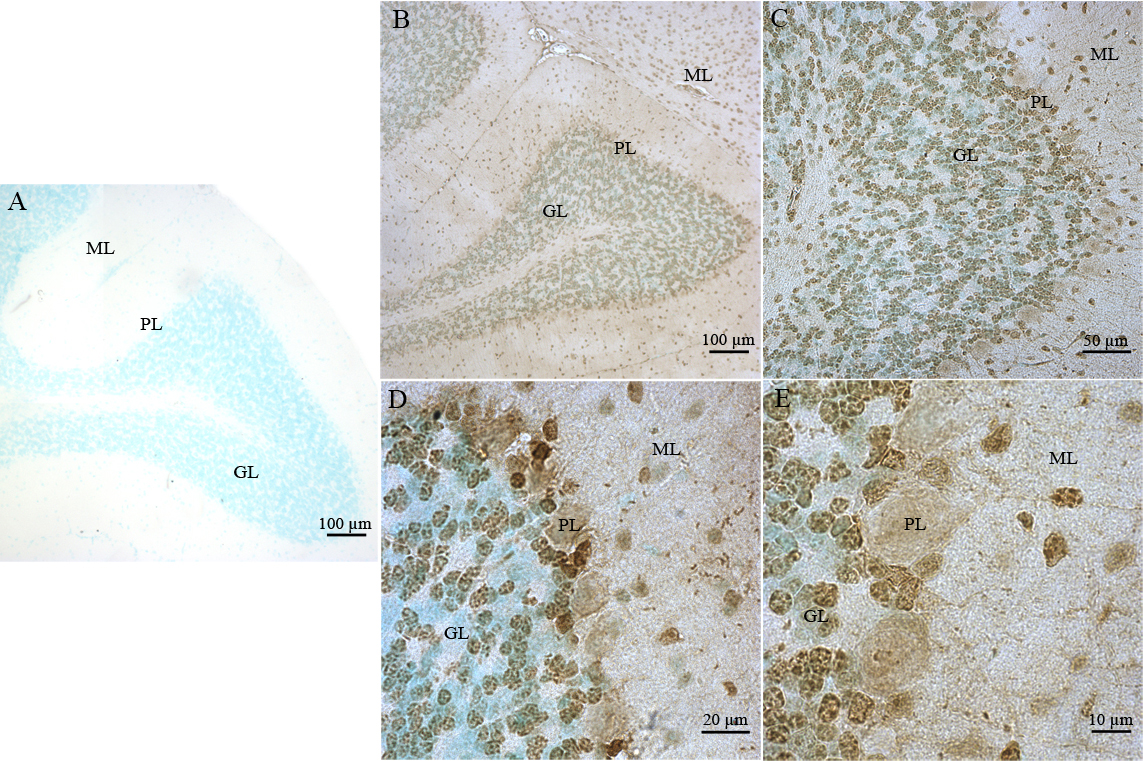
Immunohistochemical localization of ATM in neurons of adult mouse cerebellar tissue sections. No specific immunostaining was detected in control experiments, where the specific anti-ATM primary antibody was omitted (**A**). ATM immunolabeling was detected in specific neuronal layers of the adult mouse cerebellum (**B**). A high magnification of ATM immunostaining in the granular cell (GL), molecular cell (ML), and Purkinje cell (PL) layers (**C-D**) is shown. A higher magnification of ATM immunostaining in the PL (**E**) is also shown.

In cerebellum tissue sections, we detected ATM immunoreactivity in the nuclei of the GL and ML of the cerebellar cortex (Figure 9B,C). Faint but significant ATM immunolabeling was observed in the nuclei of the PL (Figure 9D,E). No ATM immunostaining was observed in the appropriate control experiments (Figure 9A).

### Detection of phosphorylated ATM in adult mouse ocular tissues

**Figure 10:**
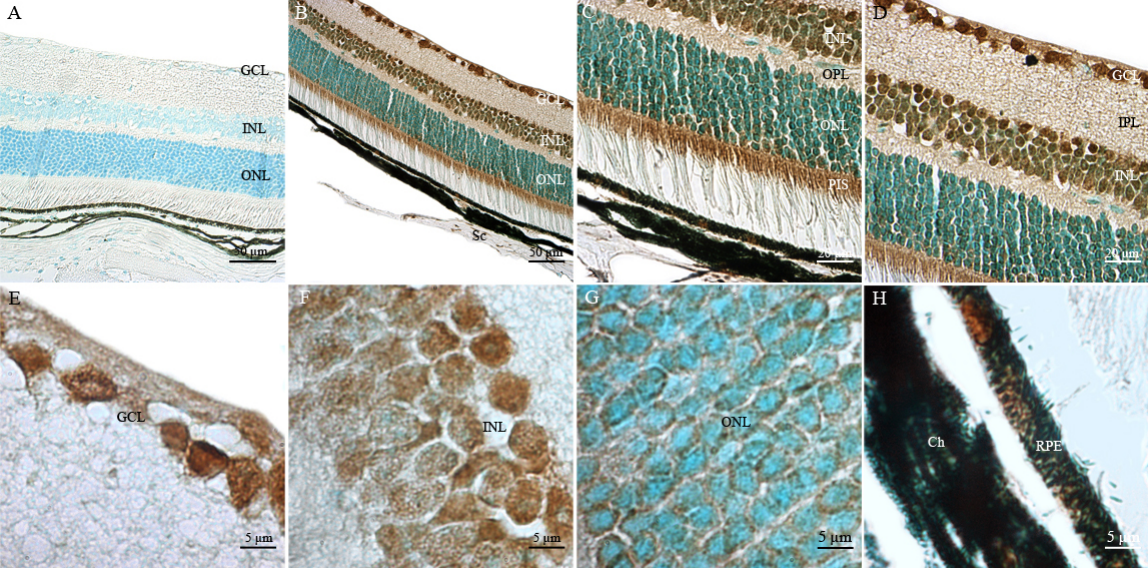
Immunohistochemical localization of ATMp in neuroretinal cells of adult mouse tissue sections. Specific mouse neuroretinal ATMp immunostaining was detected in adult mouse ocular tissue sections, using an antibody raised against ATMp (the rabbit polyclonal pS1987 antibody from Signalway was used for this figure). The same pattern was observed with rabbit polyclonal antibody pS1987 from Abcam and mouse monoclonal antibody pS1987 from Rockland. No specific immunoreactivity was detected in control experiments where the specific primary antibody was omitted (**A**). ATMp immunostaining appears with a brown color and sections were counterstained with a methyl green solution. Cellular distribution of ATMp immunostaining was observed in retinal cells (**B**). A high magnification of the outer part (**C**) and inner part (**D**) of the neuroretina is shown. A high magnification of the ganglion cell layer (GCL; **E**), inner nuclear layer (INL; **F**), outer nuclear layer (ONL; **G**), and retinal pigment epithelium (RPE; **H**) is also shown. Abbreviations: choroid cells (Ch), inner plexiform layer (IPL), outer plexiform layer (OPL), photoreceptor inner segment (PIS), and scleral cells (Sc).

Given the potential role of ATM in the maintenance of cell integrity and its broad distribution throughout the adult mouse eye, we studied the expression of its active phosphorylated form (ATMp) in adult ocular tissues using immunohistochemistry and western blot analysis. For immunohistochemistry experiments, we used three antibodies specific for mouse phosphorylated ATM at serine 1987 (pS1987; see Methods). The same pattern was observed with these antibodies. We used one of these pS1987 antibodies (Rockland) for western blotting. We detected a specific band in each lane of DNA electrophoresis migration, which corresponded to the size of ATMp. This band was not observed in *Atm*-deficient mice (Figure 6). We detected strong ATMp immunoreactivity in all cell layers—GCL, INL, ONL, IPL, and OPL—and in the PIS in adult retinal sections (Figure 10B-D). ATMp immunoreactivity showed a similar pattern to that of ATM in GCL and INL (Figure 10E,F; Figure 7E,F). ATMp seemed to be mainly, but not exclusively, detected in the nuclei of these neuroretinal layers, whereas it was mostly detected in the cytoplasm of photoreceptor cells (Figure 10G). We also detected ATMp immunostaining in the RPE cell layer and in Ch and Sc cells (Figure 10B,H). No immunostaining was observed in the appropriate control experiments performed with each ATMp antibody (Figure 10A).

These highly reproducible observations shown in Figure 10, which strongly support the spontaneous activation of ATM in both retina and cerebellum, especially in photoreceptor cells and cerebellar granule cells, combined with the detection of DNA damage - using specific 8 oxo-dG and γ-H2AX antibodies - in the same cells previously shown above in Figure 1 contribute to establish the existence of a clear link between the occurrence of high spontaneous levels of oxidative stress induced DNA lesions and ATM phosphorylation in these very cells.

**Figure 11:**
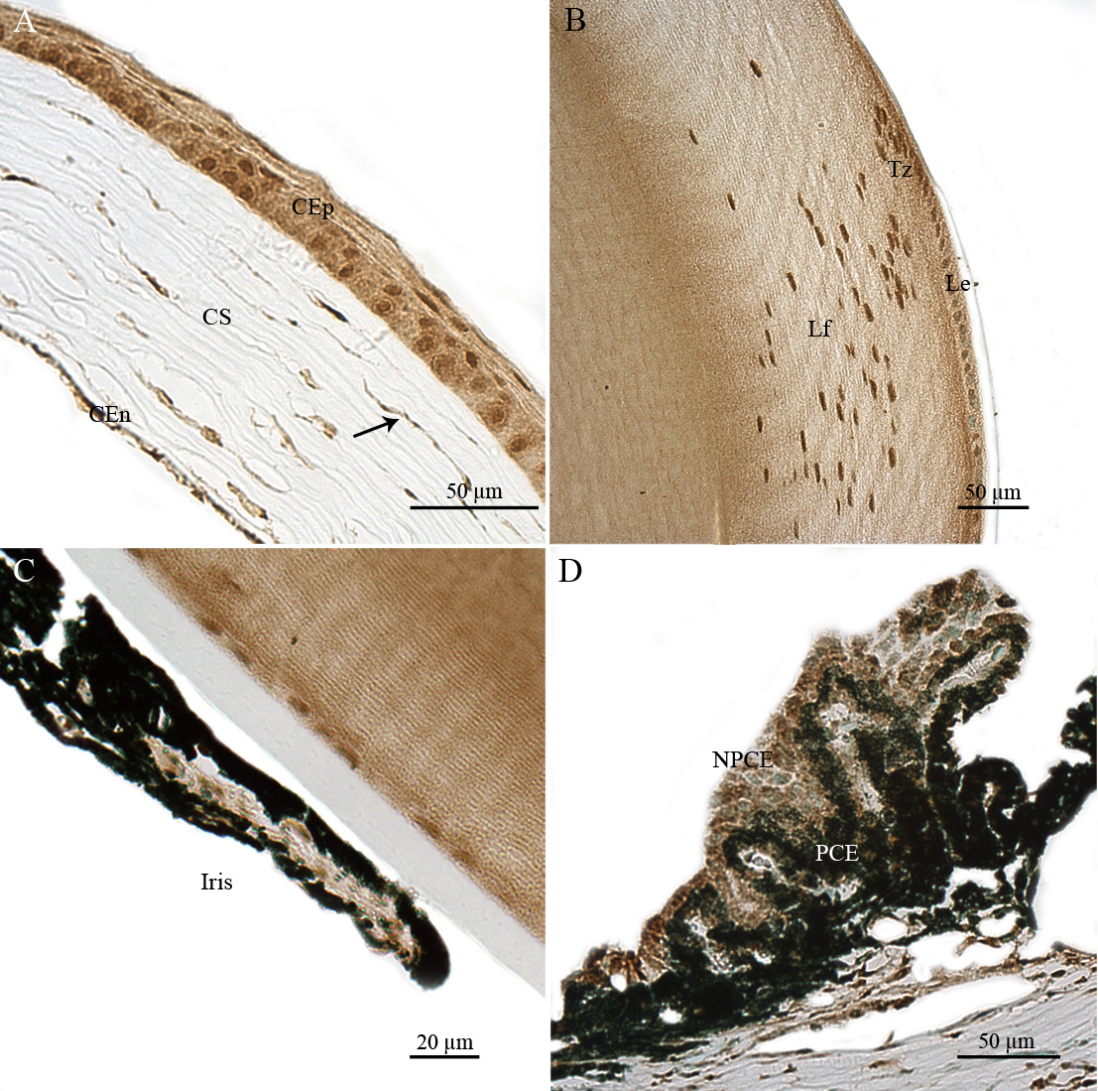
Immunohistochemical localization of ATMp in nonretinal cells of adult mouse ocular tissue sections. The rabbit polyclonal pS1987 antibody from Signalway was also used for this figure. The same pattern was observed with the rabbit polyclonal antibody pS1987 from Abcam and mouse monoclonal antibody pS1987 from Rockland. ATMp immunolabeling was observed in corneal epithelial (CEp), corneal stromal (CS), and corneal endothelial (CEn) cells, an arrow indicates the specific nuclear and cytoplasmic ATMp immunostaining of corneal cells, (**A**); in the lens epithelium (Le), transitional zone (Tz), and lens fibers (Lf; **B**); in iridal cells (**C**), in the pigmentary ciliary epithelium (PCE) and nonpigmentary ciliary epithelium (NPCE) cells (**D**).

Among the non-neuronal cells of ocular tissue sections, we observed strong ATMp immunostaining in the nucleus and cytoplasm of CEp, CS, and CEn cells (Figure 11A). Whereas ATM cytoplasmic immunostaining was faint in many corneal cells, ATMp signal was strong in both the nucleus and cytoplasm of all corneal cells. ATMp immunostaining of the keratocytes illustrates quite clearly the detection of the strong ATMp immunoreactivity, both in the nucleus and cytoplasm of these cells, with an intense and widespread ATMp immunolabeling evident throughout the cytoplasm (arrow in Figure 11A). ATMp immunostaining was observed in the nucleus and cytoplasm of Le, Tz, and elongating nucleated Lf cells (Figure 11B). We detected strong ATMp immunostaining in iridal cells, whereas a faint ATM immunoreactivity was evident in the same cells (Figure 11C). We detected ATMp immunoreactivity in PCE and NPCE cells of the ciliary body (Figure 11D). ATMp appeared to be present in the nucleus and cytoplasm in all iridal, PCE, and NPCE cells.

**Figure 12:**
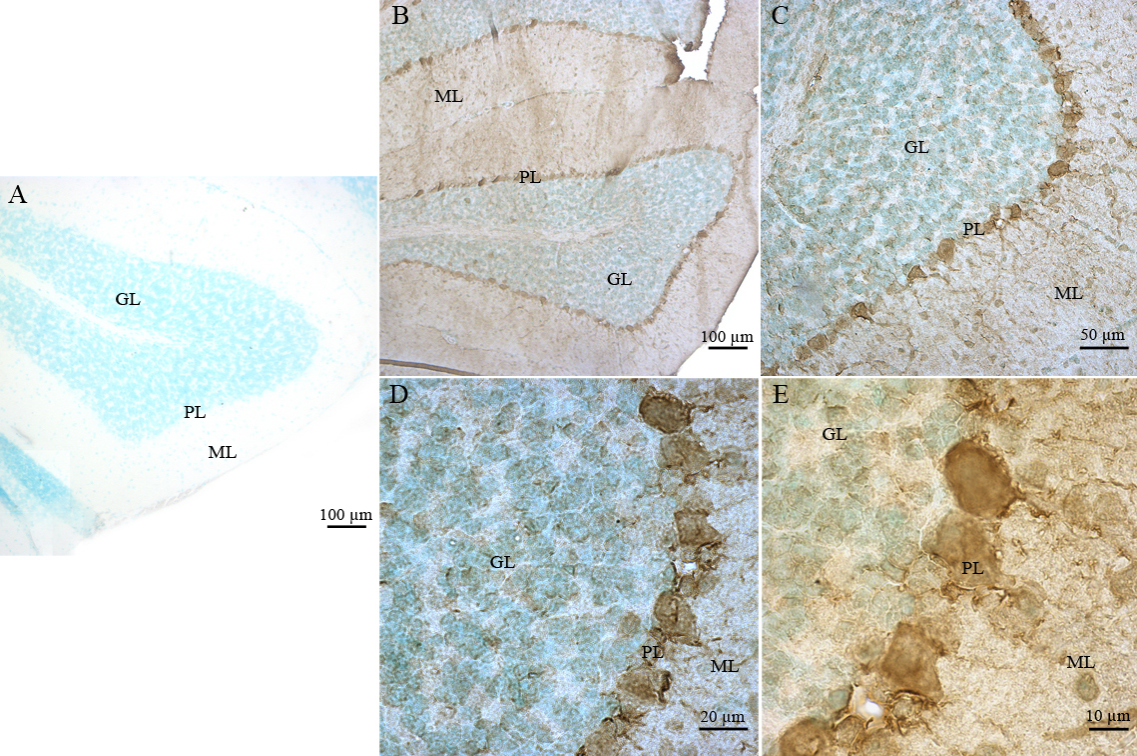
Immunohistochemical localization of ATMp in the adult mouse cerebellar tissue sections. We again used the rabbit polyclonal pS1987 antibody from Signalway for this figure. The same pattern was observed with the rabbit polyclonal antibody pS1987 from Abcam and mouse monoclonal antibody pS1987 from Rockland. No specific signal was detected in control experiments where the specific anti-ATMp antibody was omitted (**A**). ATMp immunolabeling was detected in specific adult mouse cerebellum neuronal layers (**B**). A high magnification of the granular cell (GL), molecular cell (ML), and Purkinje cell (PL) layers (**C-D**) is shown. The ATMp immunostaining of some Pukinje cells axons is clearly visible. A higher magnification of the Purkinje cell layer is also shown (PL; **E**).

We observed strong nuclear ATMp immunolabeling in the PL (Figure 12D,E), but weak immunolabeling in the cytoplasm of the GL (Figure 12B,C); this was in contrast to the nuclear immunolabeling observed for ATM (Figure 9B,C). No immunostaining was seen in the appropriate control experiments performed with each antibody (Figure 12A).

### Subcellular localization of ATM and ATMp in adult mouse photoreceptors

Our findings revealed ATM immunolabeling in the nuclei of all retinal cell layers, but a different pattern of localization was observed for ATMp. We detected ATMp immunostaining, mostly in the cytoplasm of photoreceptor cells, but mainly in the nuclei of other retinal cells. We also observed the same distinct and specific patterns of ATM and ATMp localization in the cerebellar GL. ATM protein was detected mostly in nuclei, whereas ATMp seemed to be localized in the cytoplasm of granule cells. To confirm this, we determined ATM and ATMp distribution and their subcellular localization using high-resolution confocal imaging of adult mouse retinal and cerebellar tissue sections.

**Figure 13:**
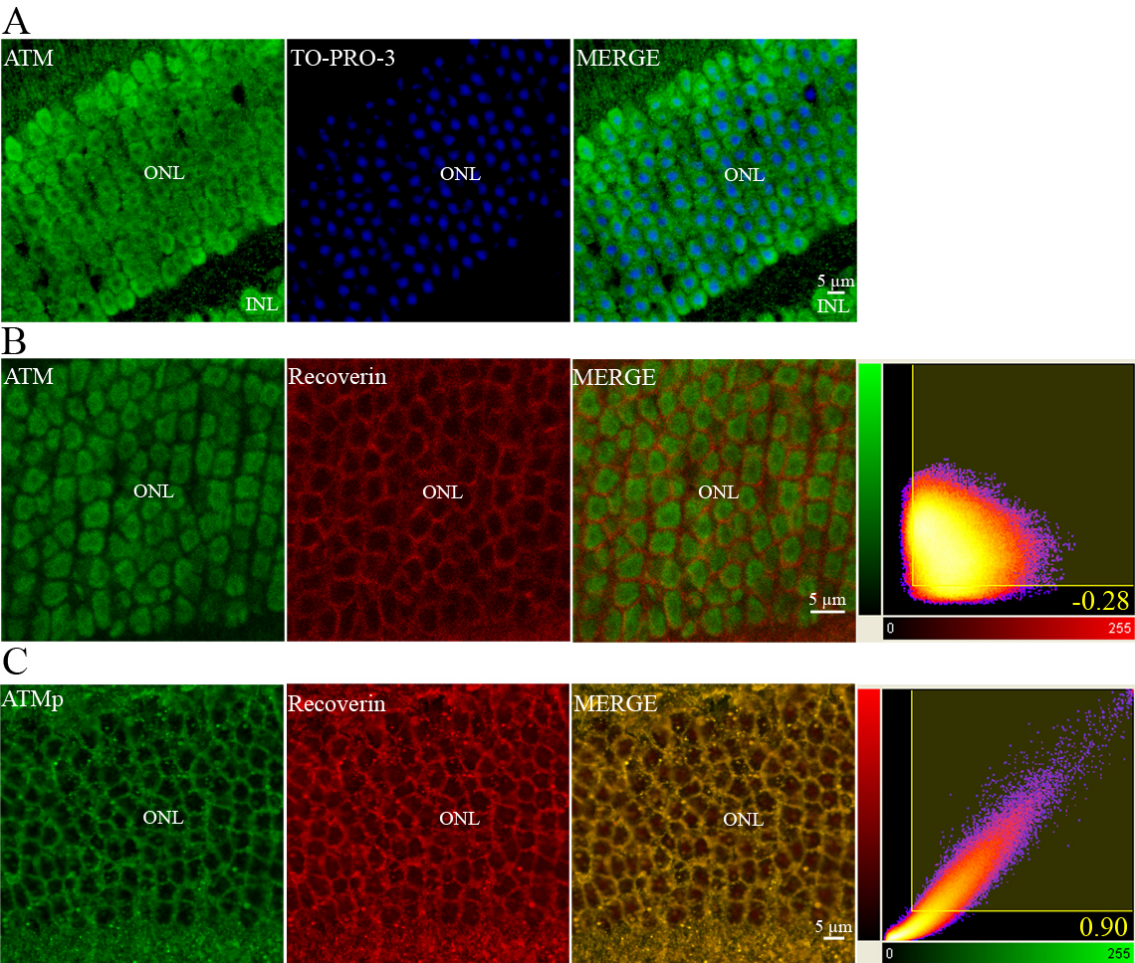
Intracellular localization of ATM and ATMp immunoreactivities in photoreceptors of adult mice using confocal imaging. The double staining of the ONL using a specific polyclonal antibody raised against ATM (green) in the neuroretina and TO-PRO-3 counterstaining (blue) is shown (**A**). The double immunostaining with anti-ATM (green) and anti-recoverin (red) antibodies is also shown. Recoverin is a specific cytoplasmic photoreceptor protein. Two-dimensional scatterplots of voxel intensities in red and green channels are shown in the right-hand column. We used a threshold of 30 (on a scale 0–255) for each label. Pearson’s correlation coefficient was determined for the correlation of voxel intensity between the red and green channels and is displayed in the lower right-hand corner. The Pearson coefficient is −0.28. No colocalization was observed between ATM and recoverin immunostainings in photoreceptor cells (**B**). The double immunostaining using an antibody raised against ATMp (green) and recoverin (red) in ONL is clearly visualized. Two-dimensional scatterplots of voxel intensities in red and green channels are shown in the right-hand column. The Pearson coefficient is 0.90. ATMp immunostaining colocalized extensively with recoverin immunostaining in photoreceptor cells (**C**). Abbreviations: Inner nuclear layer (INL), and outer nuclear layer (ONL).

Confocal imaging showed ATM immunolabeling to be present in the nuclei of the ONL. Colocalization of ATM and nuclear DNA staining (TO-PRO-3) was particularly marked at the peripheral nucleoplasmic euchromatin, which surrounds the central densely stained heterochromatin [67] (Figure 13A). We used an anti-recoverin antibody to further investigate the subcellular localization of ATM in the ONL. Recoverin is a cytoplasmic protein involved in the visual transduction cascade in both types of photoreceptor cells (cones and rods). The anti-recoverin antibody revealed a specific immunostaining exclusively in the area surrounding the nuclear region, which is immunoreactive for ATM (Figure 13B). We analyzed confocal images using the “Colocalization” module of the Imaris software package (Bitplane) to quantify the extent of ATM and recoverin colocalization. Results of the correlation analysis for the staining intensity of both antibodies were expressed as Pearson correlation coefficients. The Pearson coefficient is a number between +1 and −1, with positive values indicating a direct correlation and values near 0 indicating no correlation. A representative image of ATM and recoverin staining is shown in Figure 13B. The correlation coefficient of overlap between them was −0.28. Thus, ATM and recoverin did not colocalize. These data provide strong evidence that ATM protein is localized mostly in the nuclei of photoreceptors cells. To confirm the localization of ATMp immunolabeling in the ONL, we performed a double immunostaining for ATMp and recoverin proteins. The double staining strongly suggested that recoverin colocalized with ATMp protein in the cytoplasm of ONL (Figure 13C). A representative image of the ATMp and recoverin immunostaining is shown in Figure 13C. The Pearson coefficient in voxels with colocalization of ATMp and recoverin was 0.9, with 50% and 65% colocalization of ATMp and recoverin immunostaining (percentage of total intensity of green and red labels), respectively. There was a high degree of colocalization between ATMp and recoverin. These data provide strong evidence that ATMp protein is primarily localized in the cytoplasm of photoreceptors cells.

**Figure 14:**
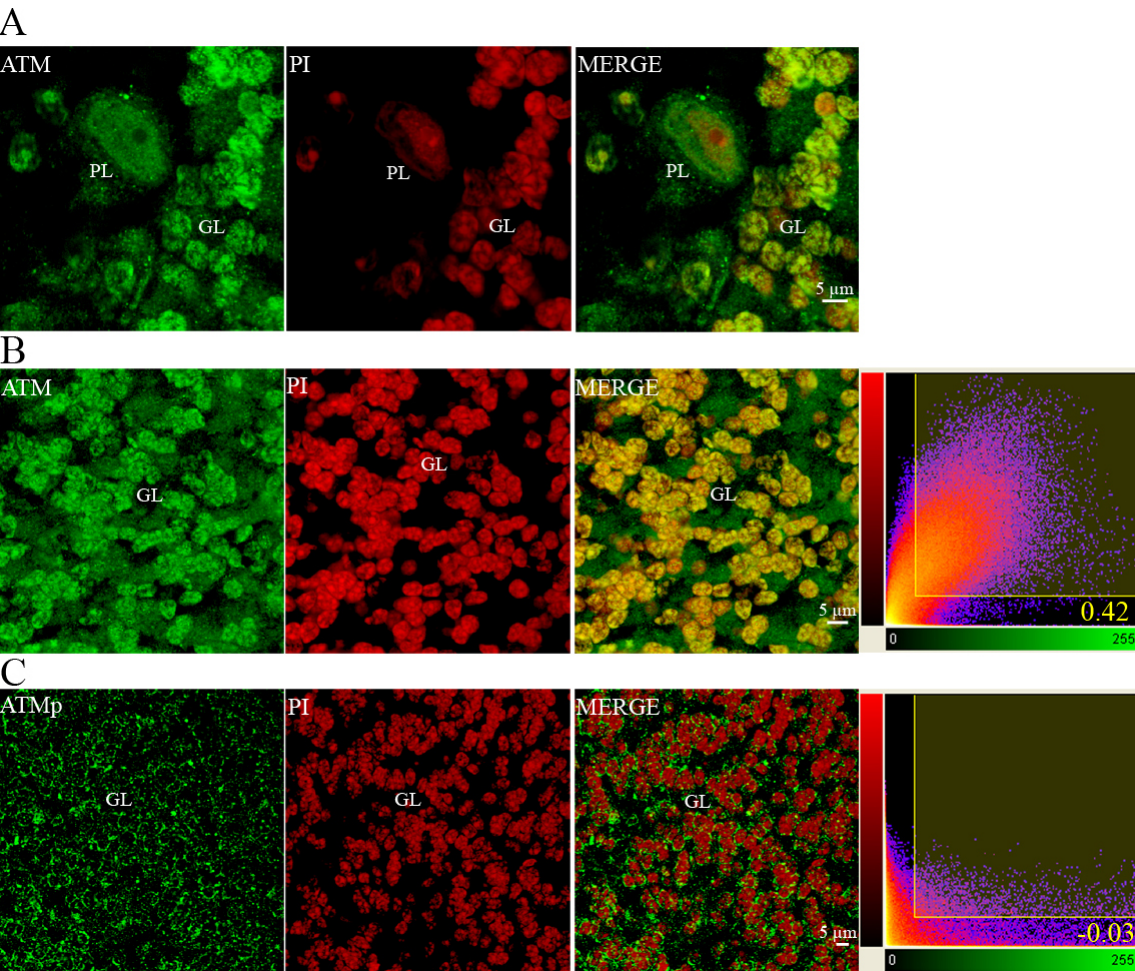
Intracellular localization of ATM and ATMp immunoreactivities in cerebellar Purkinje and granule cells of adult mice using confocal imaging. We used the monoclonal antibody pS1987 from Rockland for this figure. The same pattern was observed with rabbit polyclonal antibody pS1987 from Abcam and rabbit polyclonal pS1987 antibody from Signalway. The double labeling of cerebellar Purkinje cells with ATM immunostaining (green) and PI staining (red; **A**) is shown. The colocalization in cerebellar granule cells of ATM immunostaining (green) and PI staining (red) is shown. Two-dimensional scatterplots of voxel intensities in red and green channels are shown in the right-hand column. The Pearson coefficient is 0.42. PI staining and ATM immunoreactivity were colocalized in cerebellar granule cells (**B**). The colocalization of ATMp immunostaining (green) and PI staining (red) in cerebellar granule cells is also shown. Two-dimensional scatterplots of voxel intensities in red and green channels are shown in the right-hand column. The Pearson coefficient is −0.03. PI staining and ATMp antibody staining were not colocalized (**C**). Abbreviations: Granular cell layer (GL), and Purkinje cell layer (PL).

In cerebellar tissue sections, ATM immunostaining in PL appeared to be predominantly nuclear, with faint but detectable labeling in the cytoplasm (Figure 14A). ATM immunoreactivity was also detected in the nuclei of GL (Figure 14B). The Pearson coefficient in voxels for colocalization of ATM immunolabeling with nuclear DNA staining by propidium iodide (PI) was 0.42, with 70% colocalization of ATM immunostaining and 72% colocalization of PI staining. These data provide strong evidence that ATM protein is essentially localized in the nuclei of granule cells. ATMp immunostaining was also detected in the cytoplasm of GL (Figure 14C). A representative image of ATMp and PI staining is shown in Figure 14C. The Pearson coefficient in colocalized voxels was close to zero, showing that ATMp and PI were not colocalized. These data confirm that ATMp was mainly in the cytoplasm of granule cells.

We can summarize our findings as follows: ATM and ATMp were distributed in the same cells in all ocular compartments, with the exception of the ONL. In most, if not all, ocular compartments, ATM cellular immunoreactivity was detected primarily in the nucleus. ATMp cellular immunoreactivity was also detected primarily in the nucleus in most ocular cells, but was also detected in the cytoplasm. Indeed, we observed ATMp immunoreactivity mostly, if not exclusively, in the cytoplasm of all photoreceptors, in the PIS layer, as well as in the OPL and in the IPL. ATM appeared to be localized exclusively in the nucleus of these cells. These contrasting distribution patterns for ATM and ATMp were also observed in the granule cells of the cerebellum.

## Discussion

The damaging effects of visible and UV radiation on the mammalian retina can be detected as functional, morphological, or biochemical changes in the photoreceptor cells [68] and RPE cells, which are especially marked with aging [69,70]. DNA alterations caused by oxidative damage in the retina under normal conditions, during aging or in retinal degenerative disorders, are likely to be detected by the same sensor systems operating elsewhere in the CNS [71]. We performed in this study the unequivocal detection of 8-oxo-dG in 2-month-old adult C57BL/6J mouse retina submitted to normal conditions of illumination, as well as the indisputable detection of 8-oxo-dG in normal cerebellar neurons, especially in granule cells. The detection of this abnormally oxidized base in these highly oxygenated cells likely reflects the oxidative stress striking many of their compartments, including mitochondrial and genomic DNA. Moreover, we did not detect any differences in the γH2AX retinal and cerebellar immunohistochemical pattern between *Atm* knockout and age-matched wild-type control mice. The technical approach used is not sensitive enough for detecting differences between *Atm* knockout and control mice. There might be compensatory systems that could make this detection extremely difficult whatever the method used. It seems plausible that ATR and DNA-PKcs serve to repair the DSBs potentially triggered by physiologic oxidative stress naturally existing in the retinal neurons in general and in the photoreceptor cells in particular. Furthermore, more detailed studies of retinal and cerebellar γH2AX phosphorylation throughout C57Bl/6J mouse lifespan are required for evaluating accurately the time course of spontaneous occurrences of DSBs and their extent both in nuclear and mitochondrial genomes. Similar studies should also be performed, both in retina and cerebellum, of *Atm^−/−^* and control mice at different stages of their lifespan, independently of any gamma irradiation.

The ATM protein acts to protect the nervous system from oxidative damage [45]. Indeed, there is substantial evidence for a role of oxidative damage in the progression of neurodegenerative diseases, including Parkinson and Alzheimer diseases [72]. *Atm*-deficient mice have both high levels of oxidative stress in cerebellum [44,73,74] and a defective DNA damage response. The cells of these mice spontaneously develop elevated levels of oxidative DNA damage [41], the cerebellar granular and Purkinje cells being particularly affected [72,75]. Additionally, cells from patients with A-T display genetic instability, hypersensitivity to radiation [34], and a continuous state of oxidative stress [31]. The A-T phenotype had been mostly attributed to a defective cellular response to DSBs and possibly also to an intrinsic defective cellular response to oxidative stress [19,31]. High levels of oxygen consumption and ROS production make the eye highly susceptible to the generation of a huge number of DNA lesions, including DSBs.

Several studies suggest that ATM protein is localized in the cytoplasm of human and murine cerebellar neurons, contrasting with its nuclear localization and functions in proliferating cells [38,39,76]. However, several teams have examined the subcellular localization of ATM and have characterized the ATM-mediated damage response in mouse cerebellar neurons [65,77-79]. These studies demonstrated that ATM is essentially nuclear in these cells, with a minor cytoplasmic fraction. Additionally, various features of the ATM-mediated damage response seen in neuron-like cells and spontaneous post-mitotic neurons are similar to those in commonly used cell lines [58]. These features include the ATM autophosphorylation, which is a hallmark of its activation, and phosphorylation of several of its downstream substrates.

We investigated the presence and distribution of ATM mRNA and protein. ATM is present in cells as two different forms: ATM and ATMp. We thus performed a comparative study of their cellular distribution in the normal adult mouse eye and cerebellum.

### ATM and ATMp expression in non-neural tissues of the adult mouse eye

The cornea and lens are the first tissues to receive and absorb the light directly. ATM immunoreactivity was mostly detected in the nucleus, with a fraction observed in the cytoplasm of all corneal epithelial cells. ATMp showed strong staining both in the nucleus and cytoplasm of all epithelial corneal cells. It has been demonstrated that *Atm* gene expression is upregulated in response to a mitogenic stimulus, resulting in an increase in ATM protein levels [66]. Moreover, during cell cycle progression in physiologic conditions, ATM is activated during mitosis and localizes at centrosomes with p53 [80]. As mitosis frequently occurs in the corneal epithelium [81], it is not surprising that both ATM and ATMp immunoreactivities are detected in these cells. Similar observations were made in all dividing cellular and fiber compartments of the adult mouse lens. ATMp immunostaining suggests the possible involvement of ATM in the differentiation and cataractogenesis of the lens. Interestingly, mice heterozygous for *Atm* gene are more sensitive than wild-type animals to the cataractogenic effects of IR [82,83]. IR-induced cataracts result from the accumulation of abnormally differentiated progeny of the lens germinative cells, which are located in the equatorial region of the lens and are in complete continuity with the lens epithelium. Moreover, the targeted disruption of *Nbs1* in the lens altering the nuclear localization of the MRN (Mre11/Rad50/Nbs1) complex contributes to the early stages of cataract development due to impaired lens fiber cell differentiation [57]. The MRN complex is essential for the cellular response to DNA DSBs and links DNA repair to activation of checkpoint signaling through the protein kinase ATM [84]. Additionally, many proteins involved in DNA repair seem to be involved in cataractogenesis [85]. Indeed, overexpression of DNA polymerase β in the lens epithelium results in the early onset of severe cortical cataracts. This mechanism seems to be linked to DNA damage of lens epithelial cells, which can result in defective cell cycle control and apoptosis. Thus, ATM is present in its activated form in lens and corneal cells to regulate cell cycle and eliminate DNA damage.

Light as well as UV radiation cross the cornea and aqueous humor of the anterior chamber to meet iridal and ciliary body cells. As may be expected, ATMp shows strong immunostaining in these cells. Indeed, the iris is a potential site of free radical damage as it is rich in polyunsaturated fatty acids, which are particularly susceptible to peroxidation [86]. Furthermore, many antioxidant enzymes, such as Cu/Zn superoxide dismutase, catalase, and acidic glutathione S-transferase are present in the iris. These enzymes are capable of coping with high amounts of ROS and other free radical species [87]. Similarly, several classical antioxidant enzymes, as well as 2-Cys peroxiredoxins (PRDX), are detected in the ciliary body [88]. These enzymes are all involved in the neutralization of hydrogen peroxide and protect the cellular components against oxidative stress. These studies suggest a physiologic function for 2-Cys PRDXs in the protection of cells in the human ciliary body [89].This suggests that ATM and ATMp are present to protect iridal and ciliary body cells from oxidative damage.

### ATM and ATMp expression in the adult mouse retina

Photoreceptor cells continuously produce high levels of ROS as a byproduct of their various functions throughout their lifespan. Thus, as may be expected, we detected ATM and ATMp immunoreactivities in these cells. The POS may be highly susceptible to lipid peroxidation by ROS due to their high concentration of docosahexaenoic fatty acyl chains and to the proximity of the likely sources of ROS production: peroxisomes and mitochondria in the adjacent PIS and RPE cells during POS phagocytosis. In this study, we detected significant levels of ATMp in PIS and in the IPL and OPL. However, the subcellular localization of ATM and ATMp in photoreceptors differed substantially from that observed in other retinal cells. Indeed, ATM immunoreactivity in the ONL was mostly, if not exclusively, confined to photoreceptor nuclei and did not colocalize with recoverin immunoreactivity. In stark contrast with ATM staining in photoreceptor cell bodies, ATMp had a predominantly cytoplasmic localization.

The function of ATM in the cytoplasm is not clear, but previous findings in mice have demonstrated that *Atm* deficiency leads to abnormalities of cytoplasmic organelles [38]. The different patterns of intracellular localization reported for ATM may result from tissue-specific functions of ATM or from cell-cycle arrest inherent to the post-mitotic nature of the cells [90]. It is widely known that cells in which ATM is localized in the nucleus undergo cell division. By contrast, neurons are post-mitotic and undergo cell-cycle arrest. It remains to be established whether the differences in intracellular localization depend on the nature of the tissue as well as the cell type producing ATM, or on any cell cycle arrest.

In the neuroretina, the activated form of ATM is present in the cytoplasm, where it may protect against oxidative damage resulting from the high levels of ROS production by peroxisomes and mitochondria-major sites of oxidative metabolism. A pioneering study suggested that some ATM protein was localized outside the nucleus in cytoplasmic vesicular structures, more precisely in the microsomal fraction [91]. Additionally, it has been demonstrated that a portion of the extranuclear ATM was localized to peroxisomes. It was indeed shown that ATM might be bound to peroxisome membranes, but ATM internalization in the peroxisomal matrix still remains to be confirmed [92]. Another study described a marked preferential distribution of ATM immunoreactivity in the endosomes of the cerebellar granule neurons, with a much lower intensity of the ATM immunohistochemical staining in the Purkinje and molecular layers [76]. Cytoplasmic ATM may be required in the mouse brain for preventing lysosomal accumulation [38]. A similar function of ATM may be required in retinal cells.

The reason for cerebellar degeneration in A-T is not clear. It has been attributed, by several investigators, to cytoplasmic functions of ATM that are not necessarily linked to the DNA damage response. PIS are highly enriched for peroxisomes and mitochondria. These organelles are sources of ROS but contain specialized antioxidant systems responsible for detoxifying reactive oxygen intermediates. However, when faced with excessively high oxidative stress and DNA damage, these systems cannot carry out their normal detoxification functions. ATM signaling is required to sense and initiate DNA DSBs repair. Therefore, nuclear genomic instability resulting from loss of this function has so far been considered as the major mechanism underlying the pathology of A-T. However, this disease presents a wide variety of symptoms, not all of which are readily explained by nuclear genomic instability. The study of cells and animal models of A-T has led to much speculation about additional pathogenic mechanisms.

Moreover, recent studies have demonstrated that ATM is involved in other important functions. Two novel intertwined roles have been proposed for ATM: the regulation of ribonucleotide reductase, the rate-limiting enzyme in the de novo synthesis of deoxyribonucleoside triphosphate, and the control of mitochondrial homeostasis [93,94]. ATM could modulate mitochondrial respiratory rates directly or indirectly, or through some DNA repair functions. Alternatively, ATM may regulate mitochondrial function in a DNA-repair-independent, hence metabolic, capacity [95]. These new findings suggest that A-T pathophysiology is more complex than anticipated and may be closely related to the field of mitochondrial diseases. The photoreceptor mitochondria are confined within the inner segment and presynaptic regions of the OPL. ATMp protein was also readily detected within the OPL of the retina, where photoreceptors form synapses with neuronal bipolar cells. Most mitochondria in the presynaptic regions of the rod bipolar neurons are concentrated in the IPL. Indeed, axonal presynaptic terminals of the bipolar cells are either synaptically connected to AII amacrine cells—which, in turn, are connected to the cone cells circuitry—whereas cone bipolar cells are directly synaptically connected to the retinal ganglion cells of the cone pathway [96]. Mitochondria are primary sites of ROS generation. Previous studies have shown that one characteristic feature of cells from patients with A-T is their state of continuous oxidative stress [31]. A-T cells display intrinsic mitochondrial dysfunction, implicating ATM in the regulation of mitochondrial function [95]. It is thus possible that the activated form of ATM reduces or prevents the deleterious effects of ROS production in the mitochondria-rich regions of retinal neurons, resulting from the normal functioning of the electron transport chain coupled with phosphorylative oxidation.

Although the highest oxygen concentrations are detected in the neural retina, one should not underestimate the importance of oxygen consumption and ATP production and thus the high amounts of ROS physiologically produced in RPE cells [97,98]. ATM protein showed immunostaining in the nucleus of RPE cells, and strong ATMp labeling was observed in these cells. The RPE plays a central role in the physiology of the retina [99]. RPE has numerous functions extremely important for the neural retina. It regulates the ionic environment of the subretinal space. It phagocytoses shed outer segments of photoreceptors. It converts the 11 *trans* retinal to the photosensitive 11 *cis* retinal, which is a key molecule of the visual cycle and phototransduction cascade. It also constitutes the outer hemato-ocular barrier of the eye. In addition to its light absorption properties, which generate free radicals, RPE is exposed to high oxygen concentrations, which may promote the generation of ROS, inducing oxidative damage in the event of impaired antioxidant defense mechanisms [68]. Indeed, POS phagocytosis subjects RPE cells to an oxidative event of the same order of magnitude as that measured in macrophages [100]. The event is not an extracellular macrophage-type respiratory burst, but may be due to intracellular hydrogen peroxide resulting from NADPH oxidase activity in the phagosome or from β-oxidation of ROS lipids in peroxisomes [101]. The significant levels of ATMp immunoreactivity observed in RPE cells may prevent the detrimental consequences, in particular DNA damage, caused by the frequent occurrence of oxidative events during physiologic RPE functions.

### Distribution of ATM and ATMp in photoreceptor and cerebellar granule cells

The cytoplasmic localization of ATMp in photoreceptors is intriguing, as some form of retinal degeneration or dysfunction would be expected to occur in *Atm*-deficient mice and A-T patients. So far, no such abnormalities have been reported. It remains to be determined why distinct ATM and ATMp immunostaining patterns are observed in granule cells and photoreceptors but not in other neuronal cells.

One possible explanation for this discrepancy may be related to the specific neuronal cell types targeted in various neurodegenerative diseases involving either the cerebellum or retina, or both. Indeed, studies performed on postmortem cerebellum samples from A-T patients unequivocally demonstrate that cerebellar granule cells are major targets of the A-T neurodegenerative process [102]. Whatever the molecular genetics cause might be for any type of inherited retinal degenerations, photoreceptor or RPE cells are the major retinal cell types that undergo degeneration (RetNet). This highlights a specific susceptibility of these cells to oxidative stress and DNA damage. The molecular basis of this cell type-specific susceptibility remains to be elucidated. The Harlequin mutant mouse strain (Hq) is an important mouse model, combining both cerebellar granule and photoreceptor cell degeneration [103]. This model has enabled the establishment of a link between oxidative damage, cell cycle reentry, and cell death. Reduced levels of apoptosis-inducing factor (Aif) were obtained in this mutant by proviral insertion. The cerebellar neurons and retinal cells in this mouse thus display increased ROS-induced DNA damage accompanied by the appearance of S-phase markers, preceding apoptotic death, cell cycle activation in neurons, and DNA synthesis. The particular vulnerability of cerebellar granule and photoreceptor cells to ROS cannot be fully understood without consideration of the crucial functions of ATM in cell survival. The consequences of ATMp cytoplasmic localization in these cell types open up new possibilities in the study of ATM-associated molecular pathways in cell survival, both in physiologic conditions and in neurodegenerative disorders. Notably, the links between the cytoplasmic localization of ATMp and the major role played by mitochondria and peroxisomes in ROS production, including vicious circles of ROS overexpression in neurodegenerative processes, have not been clearly established.

Another alternative, but not exclusive, explanation of these distinct patterns of ATM and ATMp localization and their possible link to the apparent sparing of photoreceptor cells in A-T patients might be provided by findings obtained in the mutant Purkinje cell degeneration mouse model (*pcd*) [104]. *Pcd* mutant mice undergo selective degeneration of specific neuronal populations: cerebellar Purkinje cells, photoreceptor cells, and olfactory mitral cells. A recent study has identified predegenerative changes, including DNA damage/repair foci in target neurons with a well preserved general cytology, in the early stages of the neurodegenerative process in these mice [105]. The substantial vulnerability of photoreceptors in *pcd* mice contrasts with the apparent resistance of the same cells in *Atm*-deficient mice. Indeed, no retinal degeneration or dysfunction has been reported so far both in A-T patients and in *Atm*-deficient mice [106-108]. However, A-T patients and *Atm*-deficient mice ophthalmic phenotypes must nowadays be reassessed by emerging noninvasive techniques increasingly available for exploring both human and mouse retinal structure and functions. This is mandatory for eliminating the possibility that A-T patients and *Atm*-deficient mice photoreceptors might actually display some subtle or manifest retinal dysfunctions corresponding to predegenerative alterations possibly reversible, such as those detected at the earliest stages of the neurodegenerative process occurring in the olfactory mitral cells of *pcd* mutant mice. Most A-T patients are affected by oculo-motor abnomalities. These abnormalities prevent in most cases the stable fixation of any visual stimulus. The absence of fixation often does not allow the ophthalmologists to perform complete and accurate visual examinations. Many A-T patients display a characteristic oculomotor apraxia (difficulty in the initiation of voluntary eye movements) frequently preceding the development of telangiectases [109]. Moreover, many A-T patients present a strabismus and/or a nystagmus [106]. It is important to emphasize that a nystagmus presumed to be of oculomotor origin might well be a consequence of retinal dysfunction or degeneration.

Another possible explanation for the discrepancy observed between ATM and ATMp cellular immunostaining patterns may involve the second master regulators of the DNA damage response: ATR protein kinase [110]. Most ATM substrates can also be phosphorylated by ATR, and the major functions of ATR and ATM in cell-cycle control are overlapping and redundant. However, ATM and ATR respond to different types of DNA damage: ATM responds to DSBs, and ATR responds to replication stress [111]. Although ATR is primarily a replication stress-response kinase, it is also activated by DSBs. A major established fact must be emphasized: both ATM and ATR are interdependent [112,113]. In A-T cells, shared ATM and ATR substrates are not phosphorylated efficiently in response to IR because ATM is absent, causing a delay in end resection. Eventually, ATM-independent end resection does occur, allowing ATR to recognize the damage [114]. The consequence of this interdependency is that *Atm*-deficient cells have severe cell-cycle checkpoint defects in response to IR exposure. The overlapping and redundant functions of ATM and ATR, as well as their specific distinct functions, may vary according to neuronal cell type. Further experiments based on conditional knockout mice for ATR as well as ATM are needed to test this hypothesis.

The importance of maintaining genomic stability in neurons cannot be overemphasized. Their finite number, long life, high metabolic rate, and continuous exposure to oxidative stress, together with high levels of gene transcription, require stringent control of genomic integrity. Our findings show that ATM is present in the adult mouse whole cerebellum and retina. ATM and its activated form seem to be required for preventing the accumulation of oxidative damage in the eye, especially in the photoreceptors of the retina. The ATM/ATMp system is involved in the protection from abnormal ROS production and irreparable lethal DSBs and DNA deletions. Retinal antioxidants and DNA repair systems seem to be particularly efficient in the adult retina; indeed, retinal malignancy is rare in adult patient retinas despite daily exposure to UV and visible light radiations and metabolism-induced damage. Our study provides further insight into the molecular building blocks and pathways underlying the highly efficient retinal systems protecting against oxidative stress, DNA damage, retinal degeneration, and malignancies.
